# Interacting cells driving the evolution of multicellular life cycles

**DOI:** 10.1371/journal.pcbi.1006987

**Published:** 2019-05-14

**Authors:** Yuanxiao Gao, Arne Traulsen, Yuriy Pichugin

**Affiliations:** Max Planck Institute for Evolutionary Biology, August-Thienemann-Str. 2, 24306 Plön, Germany; University of California Irvine, UNITED STATES

## Abstract

Evolution of complex multicellular life began from the emergence of a life cycle involving the formation of cell clusters. The opportunity for cells to interact within clusters provided them with an advantage over unicellular life forms. However, what kind of interactions may lead to the evolution of multicellular life cycles? Here, we combine evolutionary game theory with a model for the emergence of multicellular groups to investigate how cell interactions can influence reproduction modes during the early stages of the evolution of multicellularity. In our model, the presence of both cell types is maintained by stochastic phenotype switching during cell division. We identify evolutionary optimal life cycles as those which maximize the population growth rate. Among all interactions captured by two-player games, the vast majority promotes two classes of life cycles: (i) splitting into unicellular propagules or (ii) fragmentation into two offspring clusters of equal (or almost equal) size. Our findings indicate that the three most important characteristics, determining whether multicellular life cycles will evolve, are the average performance of homogeneous groups, heterogeneous groups, and solitary cells.

## Author summary

Multicellular organisms are ubiquitous. But how did the first multicellular organisms arise? It is typically argued that this occurred due to benefits coming from interactions between cells. One example of such interactions is the division of labour. For instance, colonial cyanobacteria delegate photosynthesis and nitrogen fixation to different cells within the colony. In this way, the colony gains a growth advantage over unicellular cyanobacteria. However, not all cell interactions favour multicellular life. Cheater cells residing in a colony without any contribution will outgrow other cells. Then, the growing burden of cheaters may eventually destroy the colony. Here, we ask what kinds of interactions promote the evolution of multicellularity? We investigated all interactions captured by pairwise games and for each of them, we look for the evolutionarily optimal life cycle: How big should the colony grow and how should it split into offspring cells or colonies? We found that multicellularity can evolve with interactions far beyond cooperation or division of labour scenarios. More surprisingly, most of the life cycles found fall into either of two categories: A parent colony splits into two multicellular parts, or it splits into multiple independent cells.

## Introduction

The evolution of multicellular life cycles is one of the most challenging questions of modern evolutionary biology. In the history of life, multicellular organisms have independently originated at least 25 times from unicellular ancestors [1]. From the very beginning, multicellular life has been shaped by interactions between different cells within heterogeneous groups [2, 3]. The role of these interactions in the emergence (or prevention) of multicellularity is an open question. Recently, there has been a rising interest in the evolution of life cycles including multicellular stages from both experimentalists [4–8] and theoreticians [9–15]. Multicellular clusters can emerge either as the result of clonal development (staying together in terms of [10]) or by aggregation of cells and smaller clusters (coming together). In the present study, we focus on competition between various “staying together” life cycles. The life cycle that leads to the fastest population growth would eventually dominate the population. We address how interactions between different cells within heterogeneous groups affect the growth competition between unicellular and multicellular life cycles. When interactions between different types of individuals within one group accelerate growth, more complex forms of multicellularity are expected to evolve in the long run.

We design our study with two specific scenarios of interacting cells in mind: the threat of free-riders in groups relying on cooperation and division of labour between cells. The very first multicellular organisms are commonly suggested to be composed of similar cells as suggested by fossils [16, 17] and experimental studies [5–7]. Cooperation between cells in these early organisms provided them benefits unavailable to solitary cells. However, free-riders gaining the cooperation benefits without paying any costs have an evolutionary advantage over cooperators, which in turn may violate the integrity of an organism [18–20]. One of the most efficient ways of policing free-riders is reproduction via single cell bottleneck, where an organism grows from a single cell. This suggests that interactions between cooperators and free-riders promote group reproduction with unicellular propagules.

The second scenario where cell interactions could play a significant role, emerges once undifferentiated multicellularity has been established and cells begin to specialise on various tasks. For example, consider filamentous cyanobacteria. During nitrogen depletion, cells in the filaments occasionally differentiate into nitrogen-fixating heterocysts that obtain sugars from neighbouring photosynthetic cells and, in turn, provide these cells with nitrogen. These heterocysts suffer a significant penalty to their own fitness, but are essential to the survival of the colony as a whole [21]. A group reproduction mode preserving the necessary association between photosynthetic and rare nitrogen fixating cells would contribute a lot to the sustainable growth of this species. Naturally, the reproduction of cyanobacteria occurs by fragmenting the parental filament into shorter multicellular chains through programmed cell death [22], so newly emerged multicellular colonies are likely to contain heterocysts and benefit from the division of labour from the very beginning. This suggests that the division of labour promotes group reproduction modes with multicellular offspring groups.

While there is no clear experimental evidence that the evolution of reproduction modes can be influenced by the interaction between cells of different types, such a hypothesis deserves close attention. There is a range of previous models investigating the evolution of the division of labour [23–26]. However, these models incorporate a single predetermined reproduction mode, or a small hand-picked set of these. The evolution of cooperation in early multicellularity gained more attention [27–29]. Given that reproduction via single cell bottlenecks is a natural policing mechanism, some aspects of the evolution of reproduction modes have been considered before. Examples are the evolution of propagule size [30, 31], as well as the comparison between the formation of cell clusters and unicellular life [32]. However, the spectrum of possible interactions between cells goes way beyond specific scenarios of cooperation and the division of labour, so this topic remains largely unexplored.

In our study, we utilise the framework developed in [15], in which a reproduction mode is considered as a way to partition the cells comprising the parent group into two or more offspring groups. Since there is always a finite number of cells in a reproducing group, there is a finite number of possibilities for group fragmentation. However, our previous study assumed that homogeneous groups were composed of a single cell type. Here, we investigate heterogeneous groups consisting of cells of two different types. Groups grow in size by means of cell division (clonal development). Upon each cell division, the cell type of newborn cells can stochastically change, so no phenotype can go completely extinct. To represent the wide spectrum of possible interactions between two types, we use a game theory approach and focus on 2 × 2 games, i.e. games in which two players with two strategies interact. The result of cell interactions are given by payoff values derived from the payoff matrix of a given game. The payoff values affect both the growth rate of the whole group as well as the different growth rates of cells within the group. The combination of the game played in a group and the fragmentation mode determines the population growth rate. By screening a wide range of fragmentation modes, we aim to find the one providing the largest growth rate, which we consider to be the evolutionarily optimal reproductive strategy for the given game. Interestingly, when group growth is independent of the group size, our model suggests that only eight life cycles can be evolutionarily optimal among all possible 2 × 2 games.

## Methods

We consider a group-structured population, where individuals of two phenotypes *A* and *B* are nested into groups. These groups incrementally grow by one cell at a time and fragment into smaller offspring groups upon reaching a critical size of *M* cells. For a given group, the time between cell divisions depends only on the size of this group and its cell composition. Thus, the growth of the group is independent of other groups and therefore at the level of groups, the population growth is density independent. Therefore, in the long run, the population converges to a stationary regime, characterised by exponential growth at a rate we call λ. As populations employing different life cycles (different critical size and/or fragmentation mode) have different growth rates, the life cycle with the largest growth rate λ will eventually take over the population.

### Cell payoff and cell division

Interactions among cells in a group are captured by a pairwise game. The game is determined by a 2 × 2 payoff matrix

$$\begin{array}{cc} & \begin{array}{cc} A & B \end{array}\\ \begin{array}{c} A\\ B \end{array} & \left(\begin{array}{cc} a & b\\ c & d \end{array}\right) \end{array},$$

where *A* gets payoff *a* or *b* from interacting with *A* or *B* respectively, whereas *B* gets *c* or *d* from *A* or B, respectively. The average payoffs are given by

$$\begin{array}{c} \alpha_{\left[i,j\right]}=\frac{(i-1)a+jb}{i+j-1}, \end{array}$$

$$\begin{array}{c} \beta_{\left[i,j\right]}=\frac{ic+(j-1)d}{i+j-1}, \end{array}$$ (1)

where *α*_[*i*,*j*]_ and *β*_[*i*,*j*]_ are the average payoff of *A* type cells and *B* type cells in a group of *i* *A*-cells and *j* *B*-cells, respectively (the −1 arises from the exclusion of self-interactions, but such self interactions have only a minor influence on our results, see S6 Appendix). Solitary cells do not play the game and their payoff is zero, so *α*_[1,0]_ = *β*_[0,1]_ = 0.

Once a cell division occurs, the probability of a cell to be chosen to divide increases linearly with its fitness, *P* ∼ 1 + *wα* if the cell is of type *A*, and *P* ∼ 1 + *wβ* if the cell is of type *B*, where *w* ≪ 1 is the selection strength and the 1 measures the background fitness identical for all cells. Therefore, the probabilities that the dividing cell will be of type *A* or *B* under weak selection, *w* ≪ 1, are

$$\begin{array}{c} P_{\left[i,j\right]}^{A}=\frac{i(1+w\alpha_{\left[i,j\right]})}{i\left(1+w\alpha_{\left[i,j\right]}\right)+j\left(1+w\beta_{\left[i,j\right]}\right)}\approx \frac{i}{i+j}+w\frac{ij}{{\left(i+j\right)}^{2}}\left(\alpha_{\left[i,j\right]}-\beta_{\left[i,j\right]}\right), \end{array}$$

$$\begin{array}{c} P_{\left[i,j\right]}^{B}=\frac{j(1+w\beta_{\left[i,j\right]})}{i\left(1+w\alpha_{\left[i,j\right]}\right)+j\left(1+w\beta_{\left[i,j\right]}\right)}\approx \frac{j}{i+j}-w\frac{ij}{{\left(i+j\right)}^{2}}\left(\alpha_{\left[i,j\right]}-\beta_{\left[i,j\right]}\right), \end{array}$$ (2)

where $P_{\left[i,j\right]}^{A}$ is the probability that some cell of type *A* will be chosen to divide in a group of *i* *A*-cells and *j* *B*-cells, and $P_{\left[i,j\right]}^{B}$ is the same for type *B*, so $P_{\left[i,j\right]}^{A}+P_{\left[i,j\right]}^{B}=1$.

Similarly, the time between two consecutive cell divisions depends linearly on the average payoff in a group

$$t_{\left[i,j\right]}=T_{i+j}\left(1-w\frac{i\alpha_{\left[i,j\right]}+j\beta_{\left[i,j\right]}}{i+j}\right),$$ (3)

where *T*_*i*+*j*_ is the size dependent component of growth, and $\frac{i\alpha_{\left[i,j\right]}+j\beta_{\left[i,j\right]}}{i+j}$ is an average payoff of cells in a group.

In our model, both $P_{\left[i,j\right]}^{A}$, $P_{\left[i,j\right]}^{B}$ and *t*_[*i*,*j*]_ are dependent on cell’s payoff. Cells with larger payoff have a higher chance (*P*_[*i*,*j*]_) to reproduce, when the group grows incrementally. Thus, also groups with larger average payoff grow faster. Otherwise, under payoff-independent growth times (*t*_[*i*,*j*]_ = *T*_*i*+*j*_), the group composition would have no effect on the group growth. Consequently, in such a case the evolution of life cycles is driven by group size alone, a scenario which we investigated in previous work [15]. In other words, selection acts on the cell level via *P*_*ij*_ and selection acts on the group level via *t*_[*i*,*j*]_.

When a cell divides, each of the two daughter cells may independently change their type with probability *m*. Thus, the daughter cells of an *A* cell are either two *A*-cells with probability (1 − *m*)^2^, or one *A* and one *B* cell with probability 2*m*(1 − *m*), or two *B*-cells with probability *m*^2^.

Once the group reaches the critical group size *M*, it immediately fragments into smaller pieces and all cells are randomly assigned to offspring groups. The life cycle is determined by the critical size *M* and the sizes of offspring groups. For instance, at *M* = 3, there are two possible life cycles: either split into three solitary cells (life cycle 1+1+1), or into a solitary cell and bi-cellular group (life cycle 2+1). For *M* = 4, there are four possible life cycles: 3+1, 2+2, 2+1+1 and 1+1+1+1. Below, we refer to different life cycles using partitions of integer numbers.

### Population growth rate

We assume that the population is able to grow without any bounds. For our model, the density of groups follows a linear differential equation and growth is exponential [15]. Our goal here is to find the overall population growth rate λ.

To do so, we need to take into account the stochastic nature of group development in our model. There are three sources of stochasticity: (i) the choice of the cell to divide, (ii) the phenotype of daughter cells after cell division, and (iii) the assignment of cells to offspring groups at group fragmentation. As a consequence, groups are born different: a newborn bi-cellular group may consist of two *A*-cells, one *A* cell and one *B* cell, or two *B*-cells. Also, due to the randomness in outcomes of individual cell divisions, initially identical groups could follow different developmental trajectories during their growth, where by “developmental trajectory”, we mean the record of all choices made among possible alternatives during the group growth.

**Fig 1:**
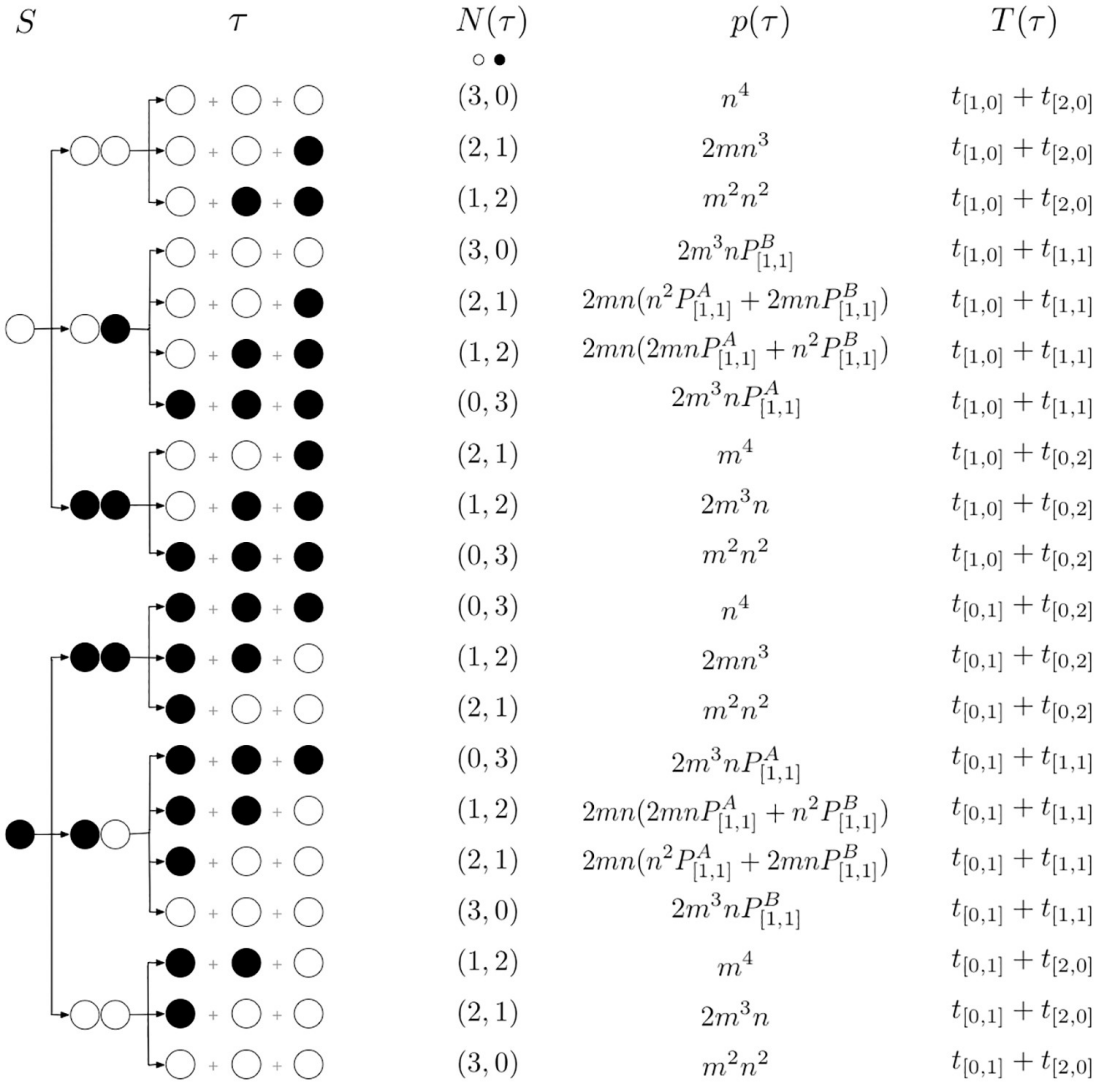
The number of developmental trajectories is finite. Here we show the full set of all 20 developmental trajectories (*τ*) in the life cycle 1+1+1, where groups are born unicellular, then grow up to size three and immediately split into independent cells. This life cycle features only two initial states *S*: solitary *A*-cell (open circles) and solitary *B*-cell (black circles). Stochastic phenotype switching creates 10 possible developmental trajectories for each initial state. To shorten the notation, we use *n* = 1 − *m* for the probability of a daughter cell to have the same phenotype as the mother cell.

Fortunately, the number of newborn states and the cell composition after each division is finite, see Fig 1. Therefore, we take all possible developmental trajectories into account for any life cycle. For an arbitrary life cycle, each group is born as one of *S* initial types, which we enumerate as (1, 2, ⋯, *S*). For each available developmental trajectory *τ*, we designate the initial state of the trajectory as *i*(*τ*), the probability that a group born at initial state *k* will follow the trajectory as *p*_*k*_(*τ*) (such that *p*_*k*_(*τ*) = 0, if *k* ≠ *i*(*τ*)), the time necessary to complete the trajectory as *T*(*τ*), and the vector of numbers of each offspring type produced at the end of the trajectory as **N**(*τ*) = (*N*_1_, *N*_2_, ⋯, *N*_*S*_).

The growth rate of population λ is given by the solution of the equation (S1 Appendix)

$$\begin{array}{c} \det(Q-I)=0, \end{array}$$ (4)

where *I* is the identity matrix and *Q* is a matrix in which

$$\begin{array}{c} Q_{i,j}\left(\lambda \right)=\sum_{\tau }p_{i}\left(\tau \right)N_{j}\left(\tau \right)e^{-\lambda T(\tau )} \end{array}$$ (5)

is the contribution of groups born as type *i* to the production of newborn groups of type *j*, see also [33].

## Results

Our model allows us to calculate the growth rate of any given life cycle provided the elements of the payoff matrix (*a*, *b*, *c*, *d*), the phenotype switching probability *m*, and the profile of the size-dependent component of development time (*T*_*i*+*j*_). Here, we focus on life cycles having the largest λ, as these will be the winners of evolutionary growth competition.

In our study, we assume that in the absence of interactions (*w* = 0), all life cycles share the same population growth rate i.e. all cells divide independently at the same rate. This assumption ensures that growth rates are exclusively determined by cell interactions. Consequently, the time of doubling the group size is the same for groups of any size. For this to be true, it is necessary to satisfy $T_{i}=\text{ln}(\frac{i+1}{i})$. Only then, the time to grow from size *k* to 2*k* is independent of *k*: $\sum_{i=k}^{2k-1}T_{i}=\text{ln}(\frac{\left(k+1)\cdot \ldots \cdot (2k\right)}{k\cdot \ldots \cdot (2k-1)})=\text{ln}\left(2\right)$. As we show in S2 Appendix, at *w* = 0 this leads to the same population growth rate λ = 1 for all life cycles. We also considered other developmental time profiles at *w* = 0 and the results of our model are similar to our previous investigation of life cycles of homogeneous groups [15], see S3 Appendix.

Under weak selection, the growth rate of the population with an arbitrary life cycle *κ* can be approximated by $\lambda \approx 1+w\lambda_{\kappa }^{\prime }$. The expression for $\lambda_{\kappa }^{\prime }$ can be obtained from a linearisation of Eq (4) with respect to *w*. Since the payoffs *a*, *b*, *c*, *d* always come into play with a factor *w* (see Eqs (2) and (3)), $\lambda_{\kappa }^{\prime }$ is linear in these payoffs. The dynamics of the population as a whole does not change if we exchange the two cell types *A* ↔ *B* and the corresponding payoff values a ↔ d, b ↔ c. Thus, *a* and *d* contribute to $\lambda_{\kappa }^{\prime }$ with the same weight and the same implies for *b* and *c*. Therefore, $\lambda_{\kappa }^{\prime }$ can be presented as a function of only three parameters: *m*, *ψ* = *a* + *d* and $\phi =\frac{b+c}{\left|a+d\right|}$. The parameter *ψ* can be interpreted as whether the formation of a homogeneous group is beneficial to the cell (*ψ* > 0) or not (*ψ* < 0), compared to a solitary cell. The value of *ϕ* is the benefit of interactions between cells of different types compared to interactions between cells of the same type. The parameter *ϕ* can also be interpreted as the benefit from the formation of a heterogeneous group compared to the formation of a homogeneous group. In a broad sense, *ϕ* captures how well groups of mixed composition perform against pure groups. The details of the calculation of $\lambda_{\kappa }^{\prime }$ can be found in the S4 Appendix.

**Fig 2:**
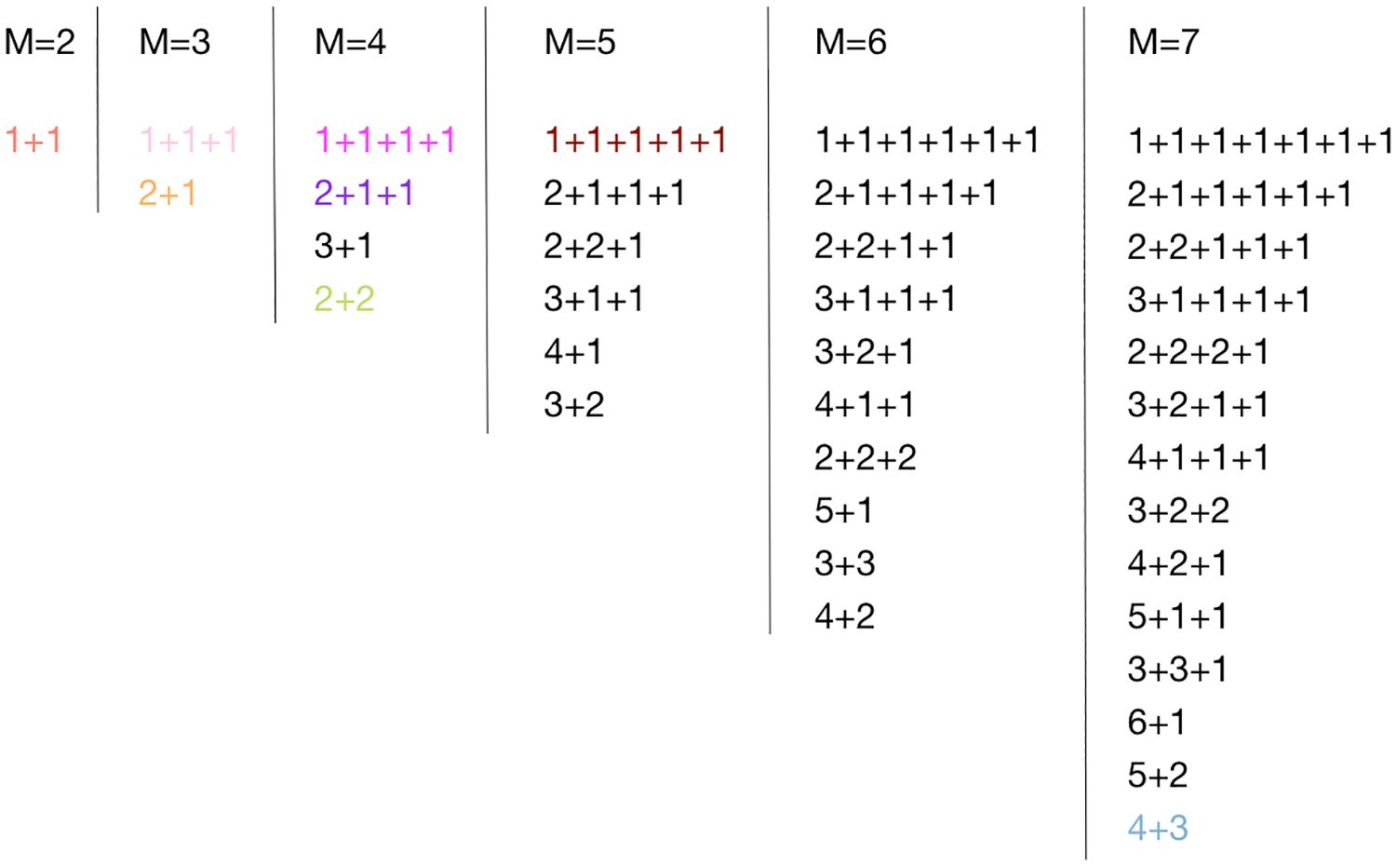
The list of all life cycles with critical sizes *M* ≤ 7. The coloured life cycles are those found to be evolutionarily optimal for some combination of the control parameters *m*, *ψ* and *ϕ*. Most life cycles were never found to be optimal. Among 24 life cycles corresponding to the two largest critical sizes *M* = 6 and *M* = 7, only one is found to be evolutionary optimal—4+3.

We numerically investigated the optimality of life cycles with fragmentation size *M* up to 7. In total, there are 37 such life cycles, see Fig 2. To illustrate the results of our approach, we begin with the presentation of evolutionary optimal life cycles for the specific case of a Prisoner’s dilemma. Consider a game with payoff matrix

$$\begin{array}{cc} & \begin{array}{cc} A & B \end{array}\\ \begin{array}{c} A\\ B \end{array} & \left(\begin{array}{cc} 1 & -3\\ c & 0 \end{array}\right) \end{array}.$$

**Fig 3:**
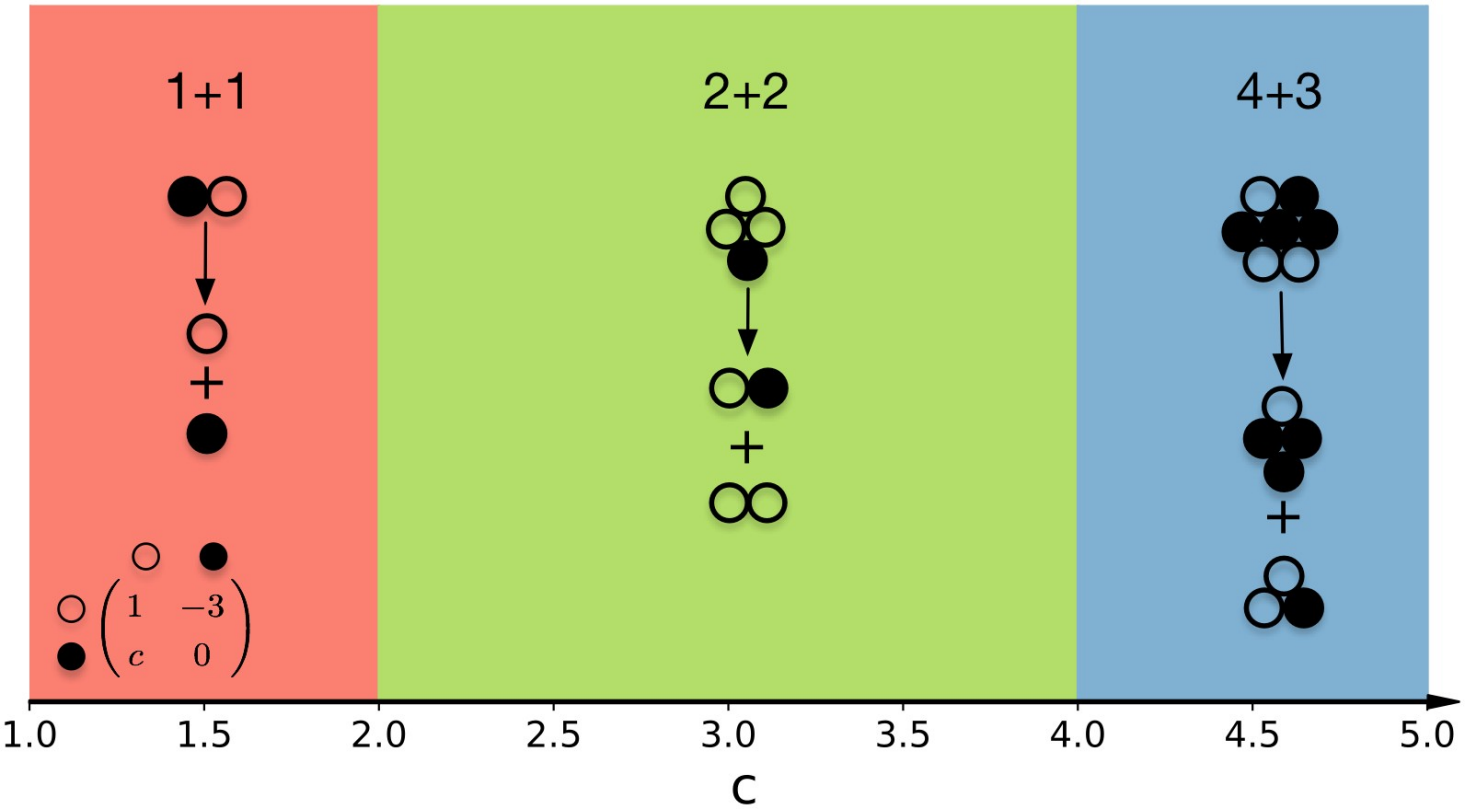
Life cycles driven by a Prisoner’s dilemma game. For *c* < 2, unicellular life cycles are optimal, as they avoid the fitness costs of heterogenous groups. For 2 < *c* < 4, the benefits of occurring in a homogeneous group compensate the risks of occurring in a heterogeneous one and the life cylce 2+2 becomes optimal. Finally, for *c* > 4, heterogeneous groups gain a larger average payoff than homogeneous groups, so the life cycle 4+3 becomes optimal. Note that the sketches of life cycles are only examples, as any distribution into black and white cells is possible (parameter values *m* = 0.5, *a* = 1, *b* = −3, *d* = 0, such that *ψ* > 0 and *ϕ* = *c* − 3).

For *c* between 1 and 5, this game is a Prisoner’s dilemma. For this payoff matrix, *ψ* = 1 and *ϕ* = *c* − 3. Additionally, we set the phenotype switching probability to *m* = 0.5. Among 37 considered life cycles, only three life cycles 1+1, 2+2, and 4+3 are found to be optimal in this case, see Fig 3. When the temptation to defect *c* is low, the best life cycle is unicellularity, as the main outcome of the emergence of defectors is merely harming the cooperators. With an increase of the temptation to defect *c*, the payoff of a heterogeneous group increases. Then, the benefits of occurring in a homogeneous group compensate the risks of occurring in a heterogeneous one. Consequently, the life cycle 2+2 becomes optimal starting from *c* = 2 (*ϕ* = −1). Finally, at large *c*, heterogeneous groups gain a larger average payoff than homogeneous groups, so the life cycle 4+3 becomes optimal at *c* > 4 (*ϕ* > 1).

**Fig 4:**
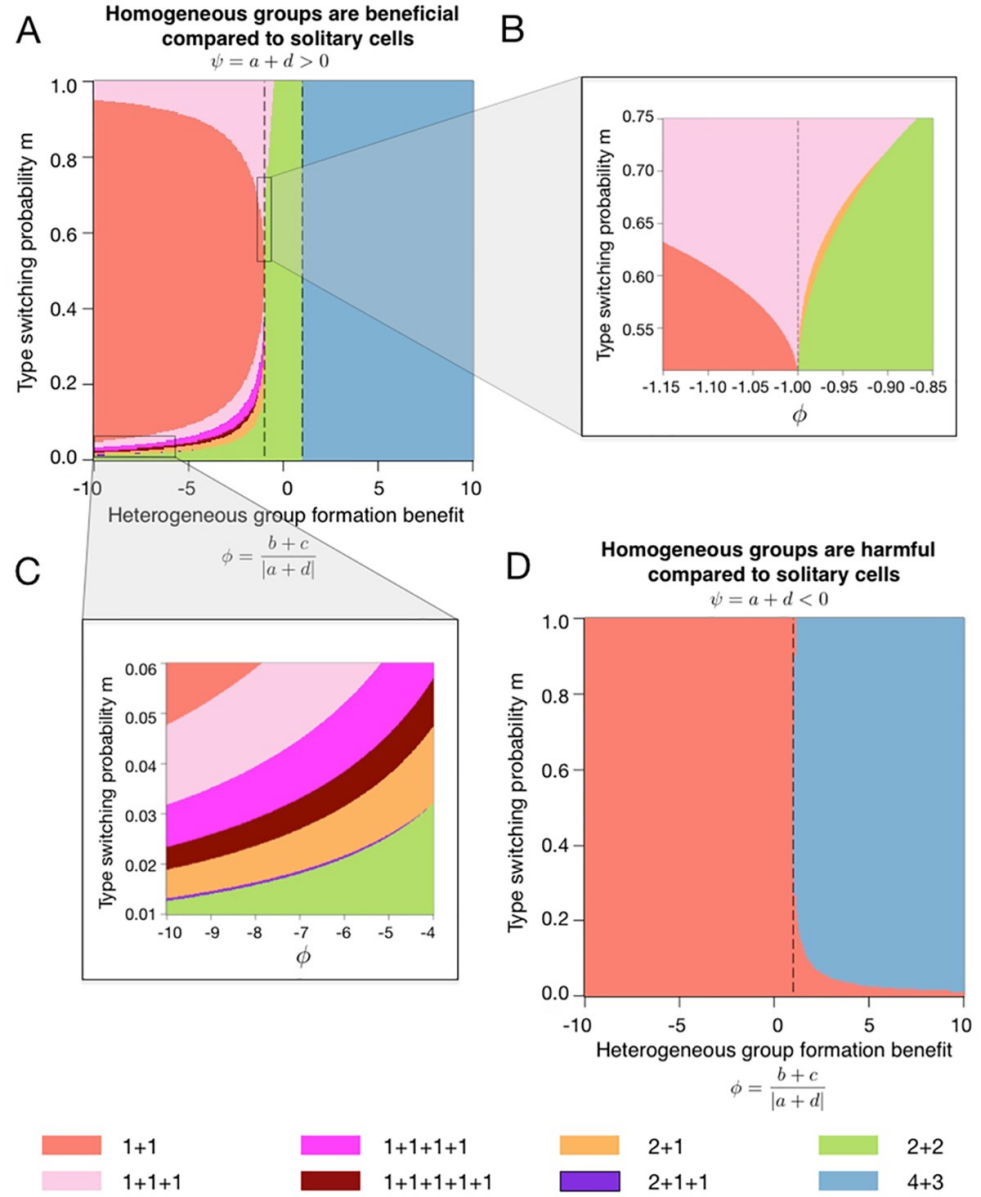
Only eight life cycles are evolutionarily optimal under weak selection for all 2 × 2 games. (A): Optimal life cycles for *ψ* > 0. Dashed lines are *ϕ* = −1 and *ϕ* = 1. (B): Enlargement of the area of large phenotype switching rate *m* > 0.5 and *ϕ* ≈ 1. In a small region within this area, the life cycle 2+1 emerges. (C): Enlargement of the area of small phenotype switching rate *m* ≪ 1, where a large diversity of life cycles is observed, including the rare life cycle 2+1+1. (D): For *ψ* < 0, only two life cycles are optimal. The dashed line is *ϕ* = 1.

Next, we proceed to the general game with arbitrary choice of each of the three control parameters *ψ*, *ϕ* and *m*. We now search for the optimal life cycle among all 37 life cycles with *M* ≤ 7. Only eight of these life cycles were found to be evolutionarily optimal for any combination of control parameters, see Fig 4. These life cycles fall into one of three categories: fission into multiple unicellular offspring (1+1, 1+1+1, 1+1+1+1, and 1+1+1+1+1); binary fragmentation with group propagules (2+2 and 4+3); and the rarely observed transition between the previous two classes (2+1 and 2+1+1).

The observed set of life cycles is affected by the limit of the maximal group size, which is only 7 cells. However, among these eight, only the life cycle 4+3 reflects this limit. Although for our methods it remains a challenge to investigate life cycles of larger groups, a clear pattern appears when we decrease the group size limit. If the group size is limited to *M* ≤ 5, the life cycle 4+3 is unavailable but the life cycle 3+2 is evolutionary optimal, instead. Extending the size limit to *M* ≤ 6, that life cycle is replaced by 3+3, and finally at *M* ≤ 7, the life cycle 4+3 takes this place. These life cycles are likely the manifestation of the more general rule “grow as large as possible and divide into two equal or almost equal parts”. Thus, for any maximal group size, we suspect that there would most likely to be only eight evolutionary optimal life cycles, seven of which would fragment to sizes five or smaller, and the eighth life cycle would be the equal split at the maximal size.

We break the remaining analysis of our results into two parts. First, we consider specific life cycles and outline the conditions which promote their evolution. Then, we take the opposite direction and focus on specific games to investigate which life cycles are promoted by them.

### Games promoting a given life cycle

First, we examine the optimal life cycles for negative *a* + *d* (*ψ* < 0), in which homogeneous groups are in adverse conditions in the first place, see Fig 4D. Consequently, one of two life cycles found here is 1+1—unicellularity, at which groups are not formed at all. Still, if *ϕ* is sufficiently large, the highest growth rate is obtained by heterogeneous groups. Then, evolutionary growth competition favours life cycles minimizing the fraction of homogeneous groups in the population. Due to the random partitioning of cells into offspring groups, smaller offspring have larger chances to accumulate cells of only one type during fragmentation. Thus, growth competition would likely promote larger offspring size to avoid such outcomes. If so, the optimal life cycle must be the fragmentation into two equal-sized (or nearly equal-sized) offspring groups at the maximal available size (4+3 in our case). Next, we focus on the more complex case of *ψ* > 0, see Fig 4A.

When *ϕ* > 1, the life cycle 4+3 is evolutionarily optimal. At these values of *ϕ*, all groups have an advantage over solitary cells, but heterogeneous groups profit more than homogeneous ones. Therefore, growth competition favours life cycles avoiding production of independent cells and minimizing the fraction of homogeneous groups in the population, i.e. an equal binary split at the maximal size. Note that at *ϕ* = 1, where *a* + *d* = *b* + *c* (equal gains from switching), there are no benefit differences between homogeneous and heterogeneous groups. As a consequence, all life cycles with multicellular offspring have the same growth rate there, see Fig 4B. The popular case of constant selection, where one type is always better off than or at least equally good as the other one, can be modelled by *a* = *b* and *c* = *d*. This implies *ϕ* = 1, and leads to the same life cycles as the more general case of equal gains from switching.

For 0 < *ϕ* < 1, 2+2 is the optimal life cycle. Here, all groups have an advantage over solitary cells, but homogeneous groups benefit more than heterogeneous ones. Therefore, growth competition would likely promote life cycles maximising the fraction of homogeneous groups in a population. First, this means producing the smallest multicellular offspring (bicellular groups) to eliminate parental heterogeneity in offspring. Second, the fragmentation has to be performed at the smallest size to minimize the risk of gaining heterogeneity in groups due to a spontaneous phenotype switch during growth. For the bi-cellular offspring, the smallest fragmentation size is four cells, therefore, the best life cycle must be 2+2. Interestingly, if *m* is small enough, the 2+2 life cycle can be optimal under arbitrary large negative *ϕ*, see Fig 4A and 4C. There, while heterogeneous groups have a strong disadvantage, chances of the phenotype switch to occur are low and homogeneity of groups is generally preserved.

At *ϕ* < 0, the emergence of another cell type in homogeneous groups incurs a penalty on the group growth. To avoid the production of heterogeneous groups, growth competition is likely to promote life cycles involving dispersal into independent cells, such that each newborn group starts in a homogeneous state.

When *ϕ* < 0 and *m* is high enough, heterogeneous groups are likely to form after the very first cell division. In this case, 1+1 is favoured as it does not involve any group formation at all. However, once *m* approaches zero, the first few cell divisions performed by initially solitary cell will likely produce a homogeneous group. Thus, multicellular life cycles with fission into independent cells are favoured: 1+1+1, 1+1+1+1, and 1+1+1+1+1, see Fig 4C and Fig 7 in S5 Appendix. Larger fragmentation sizes, first 3, 4, and then 5, become optimal with decreasing *m*. However, fission at size 6 was never found to be optimal, because at this stage, the production of multicellular offspring becomes beneficial, despite the risk of transferring parent heterogeneity into the next generation.

Transitional life cycles 2+1 and 2+1+1 are found to be optimal between areas of optimality of multiple fission life cycles (1+…+1) and multicellular offspring life cycles (2+2 and 4+3), see Fig 4C. These two life cycles mix unicellular and multicellular offspring. This may be a result of a compromise between producing multicellular offspring to fully utilize benefits of interactions in homogeneous groups, and the necessity to fragment into independent cells to purge emerging heterogeneous groups.

### Life cycles promoted by prominent games

The most prominent game in the context of evolutionary game theory is the Prisoner’s dilemma [28, 34–36]. In the simplest form of the Prisoner’s dilemma, the donation game, each player may pay some cost $\tilde{c}$, so that the opposing player will receive a benefit $\tilde{b}$ (larger than the cost). The cooperating strategy is to pay the cost, while the defecting strategy is to abstain from paying this cost (but still receive incoming benefits). The largest combined payoff is achieved by both players cooperating, while the individual’s payoff resulting from defecting behaviour is always larger than payoff from mutual cooperation. The conflict between an individual’s and group’s interests makes this game a social dilemma.

The payoff matrix of the simplest Prisoner’s dilemma is given by

$$\begin{array}{c} (\begin{array}{cc} a & b\\ c & d \end{array})=(\begin{array}{cc} \tilde{b}-\tilde{c} & -\tilde{c}\\ \tilde{b} & 0 \end{array}). \end{array}$$

With these payoffs, $\psi =\tilde{b}-\tilde{c}>0$ and $\phi =\frac{\tilde{b}-\tilde{c}}{\tilde{b}-\tilde{c}}=1$. Surprisingly, this game exhibits a special behaviour in our model: any life cycle which does not pass through the unicellular stage (e.g. 3+2+2) is evolutionarily optimal, independently of the phenotype switch probability *m* (i.e. risk of defector emergence). Contrary to our intuition, cooperative cell interactions described by the Prisoner’s dilemma promote everything *except* reproduction via the single cell bottleneck. This is due to the fact that in a group with at least one cooperator, some benefit is already produced and shared across the group. Thus, preserving group living is more advantageous for the population than producing single cell propagules.

Other notable social dilemmas are the snowdrift game and the stag hunt game. In the snowdrift game, a combined cost $\tilde{c}$ must be paid for the benefit $\tilde{b}$ to be received by each player. Cooperators readily pay their share of the costs, while defectors abstain from paying it. The payoff matrix of the snowdrift game is

$$\begin{array}{c} (\begin{array}{cc} a & b\\ c & d \end{array})=(\begin{array}{cc} \tilde{b}-\tilde{c}/2 & \tilde{b}-\tilde{c}\\ \tilde{b} & 0 \end{array}). \end{array}$$ (6)

This results in $\psi =\tilde{b}-\tilde{c}/2>0$ and $\phi =\frac{2\tilde{b}-\tilde{c}}{\tilde{b}-\tilde{c}/2}=2$. According to our findings, these parameters promote the life cycle 4+3, or, more generally, equal binary fragmentation at the maximal possible size, which ensure heterogeneous groups that maximize the combined payoff.

In the stag hunt game, players may pursue a hare—small prey providing payoff *h*, or a stag—large prey giving payoff *s* > *h*. A hare hunt is always successful, but only both hunters together can hunt down a stag. The payoff matrix of the stag hunt game is

$$\begin{array}{c} (\begin{array}{cc} a & b\\ c & d \end{array})=(\begin{array}{cc} s & 0\\ h & h \end{array}). \end{array}$$ (7)

This results in *ψ* = *s* + *h* > 0 and $\phi =\frac{h}{s+h}<\frac{1}{2}$. These parameters promote the life cycle 2+2. In contrast to the Prisoner’s dilemma and snowdrift games, in the stag hunt game a group of mixed composition has the smallest combined payoff (which is still larger than zero payoff for solitary cells for our choice of parameters). Therefore, the stag hunt game strongly favours a life cycle preserving homogeneity of groups, i.e. 2+2.

Many other evolutionary games have been studied and applied in a wide variety of biological situations [37–40]. For the case of 2 × 2 games, in a large well-mixed population of players, three classes of evolutionary dynamics are possible: dominance of one strategy (*a* > *c*, *b* > *d* or *a* < *c*, *b* < *d*), bistability (*a* > *c*, *b* < *d*) or coexistence (*a* < *c*, *b* > *d*) [34, 41].

All games experiencing a bistability (such as the stag hunt game) have *ϕ* < sign(*ψ*). According to our results, for positive *ψ*, bistability games can promote 7 out of 8 found life cycles: equal binary split at the maximal size (4+3) never leads to the fastest growth rate. For negative *ψ*, bistability games only lead to a unicellular life cycle (1+1). Games featuring coexistence dynamics (such as the snowdrift game) satisfy *ϕ* > sign(*ψ*), which restricts the optimal life cycle to 4+3 under *ψ* > 0 but allows both 1+1 and 4+3 under *ψ* < 0. Dominance games (such as the Prisoner’s dilemma) may have any value *ϕ*, so they can promote any of the 8 found life cycles.

## Discussion

In our study we performed an extensive investigation of the competition of life cycles driven by interactions between cells within in a group. Key to this study is the consideration of all possible reproduction modes and all possible interactions captured by game theoretic 2 × 2 payoff matrices. Among the huge variety of reproduction modes, only eight were found to be evolutionarily optimal, see Figs 2 and 4. Moreover, the vast majority games promotes either of two very specific classes of life cycles: fragmentation into strictly unicellular offspring (1+…+1) or production of exactly two strictly multicellular daughter groups of identical (or almost identical) size. Intuitively, life cycles with unicellular offspring should be promoted when the cells grow fast in a homogeneous group, as the single cell bottleneck eliminates heterogeneity in the most effective way. Similarly, when the cells grow fast in a heterogeneous group, life cycles with multicellular offspring should be promoted as they are best at preserving heterogeneity. Our results, in general, support this intuition. However, the current work reveals a much broader picture and we observed a number of less intuitive features of life cycle evolution driven by cell interactions. First, we observed the transition between these two major life cycles classes. This occurs via transitional life cycles mixing unicellular and multicellular offspring (such as 2+1 and 2+1+1), see Fig 4C. Second, we found that if being in a heterogeneous groups incurs a moderate penalty onto the cell, growth competition may still promote the life cycle with only multicellular offspring (2+2), even at high rates of phenotype switching (*m*), see Fig 4A. Third, an arbitrary strong penalty to heterogeneous groups (*ϕ* < 0), may still lead to the evolution of life cycles with multicellular offspring (2+2) given small enough *m*, see Fig 4A and 4C. Altogether, even with only eight life cycles observed, our model exhibits a rich behaviour and gives insights into factors shaping the evolution of life cycles.

We found that social dilemma games may not promote the evolution of single cell bottlenecks. A naive intuition suggest life cycles with unicellular offspring to be favoured by all social dilemmas, as a single cell bottleneck is an effective way to police defectors. However, social dilemmas may lead to the evolution of any of the eight life cycles. What would be the reason for such a counter-intuitive outcome?

A key difference between our approach and the most of studies utilizing evolutionary game theory is that while we allow the competition between different cell types (by means of different division probabilities *P*^*A*^ and *P*^*B*^), winning in such a competition is not in the focus of our attention. We consider both cell types as essential components of group development. This is in line with the previous idea of [9] that cheaters may play a significant role in the evolution of life cycles in early multicellularity. Embracing this approach, we acknowledge that life cycles showing the largest population growth rates are not necessarily the best in keeping cheaters out. Our results show that for evolution to favour single cell bottlenecks, a group mixing cooperators and defectors should have lower average fitness than an equivalent pack of independent cells. Otherwise, life cycles with multicellular offspring will be promoted.

This leads to a second key feature of our model: the role of solitary cells. Independent cells stand out as they have no other cell to interact with and, thus, do not play a game. As such, they serve as a benchmark of the cell behaviour, against which all other group compositions are compared. Our results indicate that optimality of life cycles strongly depends on whether a (homogeneous) group formation is beneficial or deleterious compared to a solitary cell, see Fig 4*A* and 4D, respectively. For the Prisoner’s dilemma game, a combination of a single cooperator and single defector, indeed harm the cooperator the most. However, the overall payoff to the group ($\tilde{b}-\tilde{c}>0$) is still larger than the zero cumulative payoff these cells would obtain if separated. Thus, the Prisoner’s dilemma promotes the production of the multicellular offspring in our model. The opportunity to abstain from the game (loner strategy) [42–47] is often viewed as a component of the secondary importance in evolutionary game theory models, despite its potential impact on microbial dynamics [48, 49]. For the evolution of life cycles, such an opportunity plays a central role. For any life cycle producing unicellular offspring, each member of the population passes through a developmental stage without any interaction. Also, an ultimate loner strategy, where no game is ever played, is implemented by the unicellular life cycle, which is the most basic and one of the most important reproduction modes. If we allow self-interactions, the optimality of life cycles changes insignificantly (see S6 Appendix) and even fewer life cycles, only five, can be optimal in this case.

The interplay between cell interactions and life cycles has been considered in previous studies. [30] compared the growth rate of two reproductive modes: a spore reproducer (multiple fission life cycles in our terms) and the fragmentation into same sized offspring groups. Based on the fitness effects from the colony size, they investigated the question which life cycle is good at eliminating mutations deleterious at the colony level.

An explicit connection between fragmentation modes and games played within the group was first made by [32]. There, authors focused on fragmentation modes in a form *x* + 1, and explicitly considered the 2+1 life cycle. Being focused on cooperation rather than evolution of life cycles, they discussed conditions promoting the evolution of cooperation.

The results of our model can be directly compared with our previous findings in [15] and [50], which considered the evolution of life cycles in homogeneous groups. There, for costless fragmentation (as in the present study), only binary fragmentation modes (i.e. in a form *x* + *y*) can be evolutionarily optimal. Once reproduction incurs a cost, fragmentation into multiple parts may evolve, but still some fragmentation modes remain “forbidden”, i.e. they cannot evolve under any fitness landscape (*T*_*i*_ in our terms). The set of evolutionarily optimal life cycles found in the current study is significantly different from the sets described above. Fragmentation in our model is costless, and yet we found that fragmentation into multiple parts may evolve due to the impact of cell interactions. Also, the life cycle 2+1+1, which may evolve in our model, belongs to the class of “forbidden” life cycles under costly reproduction, so it cannot evolve among homogeneous groups at all. Thus, the introduction of heterogeneity and interactions between different cell types make it possible for previously unattainable life cycles to evolve.

In our work, we adopted the minimal setup of the heterogeneous groups—colonies with two cell types. The model can be extended by considering a larger number of cell types to model more developed organisms. In such a hypothetical model, the payoff matrix is larger than 2 by 2. Consequently, the set of control parameters is larger than just (*ϕ*, *ψ*) as in the current study, so the complete analysis will be significantly more complex. Additionally, more types will require more sophisticated methods of phenotype switching than the single phenotype switching probability *m*. Naturally, in complex multicellular organisms, the phenotypes of cells are determined by developmental programs of the organism, which might be very complex.

In our model, we consider groups as a well mixed collection of cells, where an interaction between any two cells are equally likely. However, natural and experimental multicellular clusters generally have a specific geometry. For example, the multicellularity formed by *Saccharomyces cerevisiae* after selection has a roughly spherical snowflake-like shape, in which the central cells have a 76% frequency of death compared to random cell death with a probability of 6% [5]. In this snowflake-like group, central cells have more neighbours and they may have a stronger influence on cell interactions than other cells within a group. As we have shown that the interactions between cells have an impact on life cycle evolution, so must have the geometry of the group as well. However, these geometric considerations will lead to models far more complex than ours.

It is a challenging question how the interactions between different cells within an organism shape its reproduction mode. The present study demonstrates that this topic can be addressed systematically. To do so, we combine evolutionary game theory with the theory of life cycles in simple multicellular organisms. Game theory is able to capture arbitrary pairwise interactions by a payoff matrix. At the same time, the theory of life cycles represents an arbitrary reproduction mode by the partition of an integer number. These two general frameworks naturally complement each other and allow holistic investigation of life cycles of organisms with heterogeneous composition, where it is impossible to evaluate the evolution of one factor neglecting another.

## Supporting information

S1 Appendix Population growth rate in the case of stochastic developmental programs. (PDF)

S2 Appendix Existence of the neutral fitness landscape in the case of homogeneous groups. (PDF)

S3 Appendix Life cycles of homogeneous groups. (PDF)

S4 Appendix Calculation of growth rates λ for life cycles of heterogeneous groups. (PDF)

S5 Appendix Profiles of growth rates of the life cycles. (PDF)

S6 Appendix Optimal life cycles landscape under the self-interaction game. (PDF)
