# Supplementary material for: Interacting cells driving the evolution of multicellular life cycles
Source: PLoS Comput Biol. 2019 May 14;15(5):e1006987. doi: 10.1371/journal.pcbi.1006987 (PMC6534324; doi:10.1371/journal.pcbi.1006987)
Supplement: S1 Appendix — (PDF) [file pcbi.1006987.s001.pdf]

Interacting cells driving the evolution of multicellular life cycles

Yuanxiao Gao[1], Arne Traulsen[1], Yuriy Pichugin[1*]

1 Max Planck Institute for Evolutionary Biology, August-Thienemann-Str. 2, 24306 Plön, Germany

* pichugin@evolbio.mpg.de

Supporting information

S1 Appendix.

Population growth rate in the case of stochastic developmental programs. Consider a population in which each group emerges as one of S initial types. These types could be the newborn groups of different size and/or composition. With time passing, a group grows from its initial size to maturity and subsequent fragmentation. The set of growth events (cells divisions, mutations, etc) may vary from group to group. We call such an event chain "developmental trajectory" and designate it as τ. Any two groups of the same initial type may adopt different developmental trajectories for a number of reasons, such as mutations, stochastic developmental programs, or different environmental conditions. We use the following parameters of the developmental trajectory: $i(\tau)$ – the initial state of the group leading to the given developmental trajectory, $p_k(\tau)$ – the probability that a group that emerged as initial type k will follow the trajectory τ, so $p_k(\tau) = 0$, if $k \neq i(\tau)$, $T(\tau)$ – the time necessary to the newborn group to complete the trajectory τ and $\mathbf{N}(\tau) = (N_1, N_2, \cdots, N_S)$ – the vector of numbers of each offspring type produced during the fragmentation at the end of the trajectory τ.

The population features an explicit maturation component: a newborn group does not reproduce until time $T(\tau)$ has passed. Thus, to describe the population dynamics and find the population growth rate λ, it is necessary to consider the population demography. To do so, we characterize each group at each moment of time by the age parameter η. We define the age in a way that the newborn group has $\eta = 0$, while the group that reached the end of the developmental trajectory and is about to fragment has $\eta = 1$. Along the trajectory, the age increases at a constant rate equal to $\frac{1}{T(\tau)}$, i.e. the rate of ageing differs between different trajectories.

From the perspective of the population dynamics, any two groups sharing the same developmental trajectory τ and age η are identical. Thus, the state of the whole population can be described by the density function $\zeta(\tau, \eta, t)$, which shows how many groups on the developmental trajectory τ have age η at the given time t. In the stationary regime, where the fraction of groups of each type stays constant, the density function grows exponentially,

$$\zeta(\tau, \eta, t) = \rho(\tau, \eta)e^{\lambda t}, \tag{8}$$

where $\rho(\tau, \eta)$ is the stationary density distribution of groups in a population.

Within a given developmental trajectory, ageing occurs at the same rate for all groups. Therefore, the dynamics of the density function at a given age η is determined by the balance between influx of maturing younger groups and the outflux of groups becoming too old. Both processes occur with the same rate $\frac{1}{T(\tau)}$, thus the density function must satisfy the transport equation

$$\frac{\partial \zeta}{\partial t} = -\frac{1}{T(\tau)}\frac{\partial \zeta}{\partial \eta}. \tag{9}$$

Combining Eqs. (8) and (9) we get

$$\lambda\rho = -\frac{1}{T(\tau)}\frac{\partial\rho}{\partial\eta}$$

The solution of this equation is

$$\rho(\tau,\eta) = \rho_0(\tau)e^{-\lambda T(\tau)\eta}, \tag{10}$$

where $\rho_0(\tau)$ is the stationary density distribution of newborn groups with $\eta = 0$.

To find $\rho_0(\tau)$, we use the fact that each newborn organism is produced as a result of the fragmentation of some mature organism. Thus, the rate of emergence of newborn organisms in the population (j_0) is the same as the rate of production of offspring in the course of reproduction of mature organisms (j_1).

For any developmental trajectory τ, the rate of entering into the newborn state per time unit is equal to

$$j_0(\tau) = \frac{\zeta(\tau,0,t)}{T(\tau)}, \tag{11}$$

where the right hand side of the equation is the product of the number of newborn groups and the rate of ageing. The number of offspring with developmental trajectory τ is equal to the product of the total number of offspring of type $i(\tau)$ produced by all mature organisms and the probability of the offspring to adopt this developmental trajectory ($p_{i(\tau)}(\tau)$)

$$j_1(\tau) = p_{i(\tau)}(\tau)\sum_{\tau'}\frac{N_{i(\tau)}(\tau')}{T(\tau')}\zeta(\tau',1,t), \tag{12}$$

where summation is performed over all possible developmental trajectories of parent groups.

Since each produced propagule is a newborn organism, $j_0(\tau) = j_1(\tau)$. Therefore,

$$\frac{\rho_0(\tau)}{T(\tau)} = p_{i(\tau)}(\tau)\sum_{\tau'}\frac{N_{i(\tau)}(\tau')}{T(\tau')}\rho_0(\tau')e^{-\lambda T(\tau')}. \tag{13}$$

To obtain the expression connecting the population growth rate λ with parameters of developmental trajectories τ, we multiply both parts by $N_j(\tau)e^{-\lambda T(\tau)}$ (note that in general $j \neq i(\tau)$) and sum over all possible developmental trajectories

$$\sum_{\tau}\frac{N_j(\tau)}{T(\tau)}\rho_0(\tau)e^{-\lambda T(\tau)} = \sum_{\tau}p_{i(\tau)}(\tau)N_j(\tau)e^{-\lambda T(\tau)}\left(\sum_{\tau'}\frac{N_{i(\tau)}(\tau')}{T(\tau')}\rho_0(\tau')e^{-\lambda T(\tau')}\right). \tag{14}$$

We define

$$X_i = \sum_{\tau}\frac{N_i(\tau)}{T(\tau)}\rho_0(\tau)e^{-\lambda T(\tau)} \tag{15}$$

$$Q_{i,j} = \sum_{\tau}p_i(\tau)N_j(\tau)e^{-\lambda T(\tau)}, \tag{16}$$

Note that $p_j(\tau) = 0$ if $j \neq i(\tau)$.

Taking into account that $p_j(\tau) = 0$ if $j \neq i(\tau)$, Eq (14) becomes

$$X_j = \sum_i Q_{i,j}X_i. \tag{17}$$

Also in the definition of $Q_{i,j}$, the result of summation over all trajectories τ is the same as over only developmental trajectories starting from the initial state of type j, since $p_j(\tau) = 0$, if

$j \neq i(\tau)$, because an organism emerged as one type has no access to developmental trajectories originated from other types.

Eq (17) can be satisfied only if

$$\det(Q - I) = 0, \tag{18}$$

where elements of matrix Q are defined by Eq (16) and I is identity matrix. This equation allows to infer the population growth rate λ if the parameters of each trajectory are known ($i(\tau)$, $p_i(\tau)$, $\mathbf{N}(\tau)$ and $T(\tau)$). In most interesting cases, this has to be done numerically.
