# Supplementary material for: Interacting cells driving the evolution of multicellular life cycles
Source: PLoS Comput Biol. 2019 May 14;15(5):e1006987. doi: 10.1371/journal.pcbi.1006987 (PMC6534324; doi:10.1371/journal.pcbi.1006987)
Supplement: S2 Appendix — (PDF) [file pcbi.1006987.s002.pdf]

Interacting cells driving the evolution of multicellular life cycles

Yuanxiao Gao[1], Arne Traulsen[1], Yuriy Pichugin[1*]

1 Max Planck Institute for Evolutionary Biology, August-Thienemann-Str. 2, 24306 Plön, Germany

* pichugin@evolbio.mpg.de

Supporting information

S2 Appendix.

Existence of the neutral fitness landscape in the case of homogeneous groups. Consider the situation, where $w = 0$ and, therefore, the group properties depend only on the group size. A group of size i grows in size to $i + 1$ within time T_i. Here we show that if $T_i = \ln\left(\frac{i+1}{i}\right)$, all life cycles have the same growth rate $\lambda = 1$. We prove this by induction:

- The base of induction is given by Eq (4), which states that if $T_1 = \ln\left(\frac{2}{1}\right)$ and $T_2 = \ln\left(\frac{3}{2}\right)$, then $\lambda = 1$ for any life cycles fragmenting at size 3 or smaller.
- The step of induction must show that if the assumption of induction holds true for maximal size M, then under adding $T_M = \ln\left(\frac{M+1}{M}\right)$, the assumption also holds true for maximal size $M + 1$. To prove the step of induction, we only need to consider life cycles fragmenting exactly at the size $M + 1$ because life cycles fragmenting at sizes smaller than $M + 1$ have $\lambda = 1$ according to the assumption of induction.

To construct the matrix Q and find the growth rate of considered life cycles, we need to characterize the set of offspring and developmental trajectories. In an arbitrary life cycle, the fragmentation of a homogeneous group of size M results in production of offspring groups of sizes ranging from 1 to M. In total, M different types of offspring can be produced, so the size of the matrix Q is M by M. Each of the offspring will grow up to size $M + 1$ and then fragment, thus there is only one developmental trajectory for each type of offspring with $p_i(\tau) = 1$. The developmental time of the trajectory $\widetilde{T}(\tau)$ is given as the sum of incremental growth time

$$\widetilde{T}_k(\tau) = \sum_{j=k}^{M} T_j = \ln\left(\frac{M+1}{k}\right), \tag{19}$$

where k denotes the size of the newborn offspring.

An arbitrary life cycle can be characterized by the distribution of offspring sizes produced upon fragmentation N_i, where i denotes the size of offspring. By the conservation of cell number during reproduction $\sum_{i=1}^{M} iN_i = M + 1$. Therefore, according to Eq (16), for an arbitrary life cycle, the elements of matrix Q_{ij} are given by

$$Q_{ij} = N_i e^{-\lambda \ln\left(\frac{M+1}{j}\right)} \tag{20}$$

To prove the step of induction, we verify whether $\lambda = 1$ is the solution of Eq (18), with matrix Q given by Eq (20). Plugging $\lambda = 1$ into Eq (20), we have $Q_{ij} = N_i \frac{j}{M+1}$, so the Eq (18) becomes

$$\begin{vmatrix} N_1 \frac{1}{M+1} - 1 & N_1 \frac{2}{M+1} & \cdots & N_1 \frac{M}{M+1} \\ N_2 \frac{1}{M+1} & N_2 \frac{2}{M+1} - 1 & \cdots & N_2 \frac{M}{M+1} \\ \vdots & \vdots & \ddots & \vdots \\ N_M \frac{1}{M+1} & N_M \frac{2}{M+1} & \cdots & N_M \frac{M}{M+1} - 1 \end{vmatrix} = 0. \tag{21}$$

Based on the properties of determinant, we can take out the coefficients of each row and each column, then the left hand side of Eq (21) becomes

$$\frac{\prod_{i=1}^{M} iN_i}{(M+1)^M} \cdot \begin{vmatrix} 1 - \frac{M+1}{1N_1} & 1 & \cdots & 1 \\ 1 & 1 - \frac{M+1}{2N_2} & \cdots & 1 \\ \vdots & \vdots & \ddots & \vdots \\ 1 & 1 & \cdots & 1 - \frac{M+1}{MN_M} \end{vmatrix}. \tag{22}$$

For convenience, we neglect the coefficient and denote $\frac{M+1}{iN_i}$ as K_i. Thus, the determinant is

$$\begin{vmatrix} 1 - K_1 & 1 & \cdots & 1 \\ 1 & 1 - K_2 & \cdots & 1 \\ \vdots & \vdots & \ddots & \vdots \\ 1 & 1 & \cdots & 1 - K_M \end{vmatrix}. \tag{23}$$

Next we calculate the determinant by splitting the first row,

$$\begin{vmatrix} 1 - K_1 & 1 & 1 & \cdots & 1 \\ 1 & 1 - K_2 & 1 & \cdots & 1 \\ 1 & 1 & 1 - K_3 & \cdots & 1 \\ \vdots & \vdots & \vdots & \ddots & \vdots \\ 1 & 1 & 1 & \cdots & 1 - K_M \end{vmatrix} = \begin{vmatrix} -K_1 & 0 & 0 & \cdots & 0 \\ 1 & 1 - K_2 & 1 & \cdots & 1 \\ 1 & 1 & 1 - K_3 & \cdots & 1 \\ \vdots & \vdots & \vdots & \ddots & \vdots \\ 1 & 1 & 1 & \cdots & 1 - K_M \end{vmatrix} + \begin{vmatrix} 1 & 1 & 1 & \cdots & 1 \\ 1 & 1 - K_2 & 1 & \cdots & 1 \\ 1 & 1 & 1 - K_3 & \cdots & 1 \\ \vdots & \vdots & \vdots & \ddots & \vdots \\ 1 & 1 & 1 & \cdots & 1 - K_M \end{vmatrix}. \tag{24}$$

For the second part, splitting the second row, we can get

$$\begin{vmatrix} 1 & 1 & 1 & \cdots & 1 \\ 1 & 1 - K_2 & 1 & \cdots & 1 \\ 1 & 1 & 1 - K_3 & \cdots & 1 \\ \vdots & \vdots & \vdots & \ddots & \vdots \\ 1 & 1 & 1 & \cdots & 1 - K_M \end{vmatrix} = \begin{vmatrix} 1 & 1 & 1 & \cdots & 1 \\ 0 & -K_2 & 0 & \cdots & 0 \\ 1 & 1 & 1 - K_3 & \cdots & 1 \\ \vdots & \vdots & \vdots & \ddots & \vdots \\ 1 & 1 & 1 & \cdots & 1 - K_M \end{vmatrix} + \begin{vmatrix} 1 & 1 & 1 & \cdots & 1 \\ 1 & 1 & 1 & \cdots & 1 \\ 1 & 1 & 1 - K_3 & \cdots & 1 \\ \vdots & \vdots & \vdots & \ddots & \vdots \\ 1 & 1 & 1 & \cdots & 1 - K_M \end{vmatrix}, \tag{25}$$

The second term in Eq (25) is zero because the determinant has two identical columns, therefore only the first term remains. Continuing splitting the remaining rows of the first term of Eq (25), we finally obtain

$$\begin{aligned}
\begin{vmatrix} 1 & 1 & 1 & \cdots & 1 \\ 1 & 1-K_2 & 1 & \cdots & 1 \\ 1 & 1 & 1-K_3 & \cdots & 1 \\ \vdots & \vdots & \vdots & \ddots & \vdots \\ 1 & 1 & 1 & \cdots & 1-K_M \end{vmatrix} &= \begin{vmatrix} 1 & 1 & 1 & \cdots & 1 \\ 0 & -K_2 & 0 & \cdots & 0 \\ 1 & 1 & 1-K_3 & \cdots & 1 \\ \vdots & \vdots & \vdots & \ddots & \vdots \\ 1 & 1 & 1 & \cdots & 1-K_M \end{vmatrix} \\
&= \begin{vmatrix} 1 & 1 & 1 & \cdots & 1 \\ 0 & -K_2 & 0 & \cdots & 0 \\ 0 & 0 & -K_3 & \cdots & 0 \\ \vdots & \vdots & \vdots & \ddots & \vdots \\ 1 & 1 & 1 & \cdots & 1-K_M \end{vmatrix} \\
&= \cdots \\
&= \begin{vmatrix} 1 & 1 & 1 & \cdots & 1 \\ 0 & -K_2 & 0 & \cdots & 0 \\ 0 & 0 & -K_3 & \cdots & 0 \\ \vdots & \vdots & \vdots & \ddots & \vdots \\ 0 & 0 & 0 & \cdots & -K_M \end{vmatrix} \\
&= (-1)^{K-1} \prod_{i \neq 1}^{M} K_i .
\end{aligned} \tag{26}$$

Now, we look back at the first term in Eq (24), we split the second row

$$\begin{aligned}
\begin{vmatrix} -K_1 & 0 & 0 & \cdots & 0 \\ 1 & 1-K_2 & 1 & \cdots & 1 \\ 1 & 1 & 1-K_3 & \cdots & 1 \\ \vdots & \vdots & \vdots & \ddots & \vdots \\ 1 & 1 & 1 & \cdots & 1-K_M \end{vmatrix} &= \begin{vmatrix} -K_1 & 0 & 0 & \cdots & 0 \\ 0 & -K_2 & 0 & \cdots & 0 \\ 1 & 1 & 1-K_3 & \cdots & 1 \\ \vdots & \vdots & \vdots & \ddots & \vdots \\ 1 & 1 & 1 & \cdots & 1-K_M \end{vmatrix} \\
&+ \begin{vmatrix} -K_1 & 1 & 1 & \cdots & 1 \\ 1 & 1 & 1 & \cdots & 1 \\ 1 & 1 & 1-K_3 & \cdots & 1 \\ \vdots & \vdots & \vdots & \ddots & \vdots \\ 1 & 1 & 1 & \cdots & 1-K_M \end{vmatrix} .
\end{aligned} \tag{27}$$

For the second term at the right hand side of Eq (27), similar to Eq (25) in the last step, we can work out that it equals $(-1)^{M-1} \prod_{i \neq 2}^{M} K_i$. That means we can get $(-1)^{M-1} \prod_{i \neq j}^{M} K_i$ when split the j-th row. So we keep the same procedure to split the remaining rows of the first term in

Eq (27). After that, the initial determinant changes to

$$\begin{vmatrix} 1-K_1 & 1 & 1 & \cdots & 1 \\ 1 & 1-K_2 & 1 & \cdots & 1 \\ 1 & 1 & 1-K_3 & \cdots & 1 \\ \vdots & \vdots & \vdots & \ddots & \vdots \\ 1 & 1 & 1 & \cdots & 1-K_M \end{vmatrix} = \begin{vmatrix} -K_1 & 0 & 0 & \cdots & 0 \\ 0 & -K_2 & 0 & \cdots & 0 \\ 0 & 0 & -K_3 & \cdots & 0 \\ \vdots & \vdots & \vdots & \ddots & \vdots \\ 0 & 0 & 0 & \cdots & -K_M \end{vmatrix}$$
$$\begin{aligned} &+ (-1)^{M-1} \sum_{j=1}^{M} \prod_{i \neq j}^{M} K_i \\ &= (-1)^M \prod_{i=1}^{M} K_i + (-1)^{M-1} \sum_{j=1}^{M} \prod_{i \neq j}^{M} K_i \\ &= (-1)^M \left(\prod_{i=1}^{M} K_i - \sum_{j=1}^{M} \prod_{i \neq j}^{M} K_i \right) \\ &= (-1)^M \left(\frac{(M+1)^M}{\prod_{i=1}^{M} iN_i} - \frac{(M+1)^{M-1} \sum_{i=1}^{M} iN_i}{\prod_{i=1}^{M} iN_i} \right) \\ &= 0, \end{aligned} \tag{28}$$

where we used $K_i = \frac{M+1}{iN_i}$ and $\sum_{i=1}^{M} iN_i = M+1$ in the last two steps.

This proves that an arbitrary life cycle fragmenting at size $M+1$ has the growth rate $\lambda = 1$, if $T_i = \ln\left(\frac{i+1}{i}\right)$ for any $i \leq M$. This means that $T_i = \ln\left(\frac{i+1}{i}\right)$ is a neutral fitness landscape for the scenario of homogeneous groups.
