# Supplementary material for: Interacting cells driving the evolution of multicellular life cycles
Source: PLoS Comput Biol. 2019 May 14;15(5):e1006987. doi: 10.1371/journal.pcbi.1006987 (PMC6534324; doi:10.1371/journal.pcbi.1006987)
Supplement: S3 Appendix — (PDF) [file pcbi.1006987.s003.pdf]

Interacting cells driving the evolution of multicellular life cycles

Yuanxiao Gao[1], Arne Traulsen[1], Yuriy Pichugin[1*]

1 Max Planck Institute for Evolutionary Biology, August-Thienemann-Str. 2, 24306 Plön, Germany

* pichugin@evolbio.mpg.de

Supporting information

S3 Appendix.

Life cycles of homogeneous groups.

LC	τ	$N(\tau)$ ○ ○○ ○○○	$p(\tau)$	$T(\tau)$
$1+1$	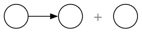	$(2,0,0)$	1	$t_{[1,0]}$
$2+1$	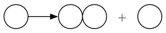	$(1,1,0)$	1	$t_{[1,0]}+t_{[2,0]}$
	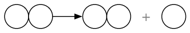	$(1,1,0)$	1	$t_{[2,0]}$
$1+1+1$	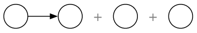	$(3,0,0)$	1	$t_{[1,0]}+t_{[2,0]}$
$3+1$	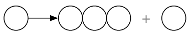	$(1,0,1)$	1	$t_{[1,0]}+t_{[2,0]}+t_{[3,0]}$
	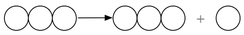	$(1,0,1)$	1	$t_{[3,0]}$
$2+2$	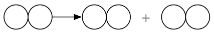	$(0,2,0)$	1	$t_{[2,0]}+t_{[3,0]}$
$2+1+1$	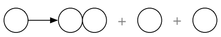	$(2,1,0)$	1	$t_{[1,0]}+t_{[2,0]}+t_{[3,0]}$
	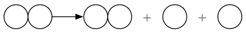	$(2,1,0)$	1	$t_{[2,0]}+t_{[3,0]}$
$1+1+1+1$	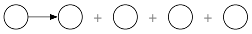	$(4,0,0)$	1	$t_{[1,0]}+t_{[2,0]}+t_{[3,0]}$

Fig 1. Homogeneous groups have deterministic developmental trajectories for each type offspring group, i.e. $p(\tau)=1$.

In the absence of cells' interactions, all cells are identical i.e. the cell type has no influence on groups. Essentially, all groups can be treated as homogeneous groups, in which only group sizes affect growth rate. In this case, groups have fixed developmental trajectories. For instance, the life cycle 1+1+1 has to go through the unique developmental trajectory: two successive divisions and then producing three single cells (see Fig 1). In this unique developmental trajectory, only one initial type exist – independent cell, so $p(\tau)=1$ and $\mathbf{N}(\tau)=3$.

First, we investigate the simplest scenario, where the maximal size of the group was limited to two cells. There are three life cycles in total in this case: 1+1, 2+1 and 1+1+1. The matrices

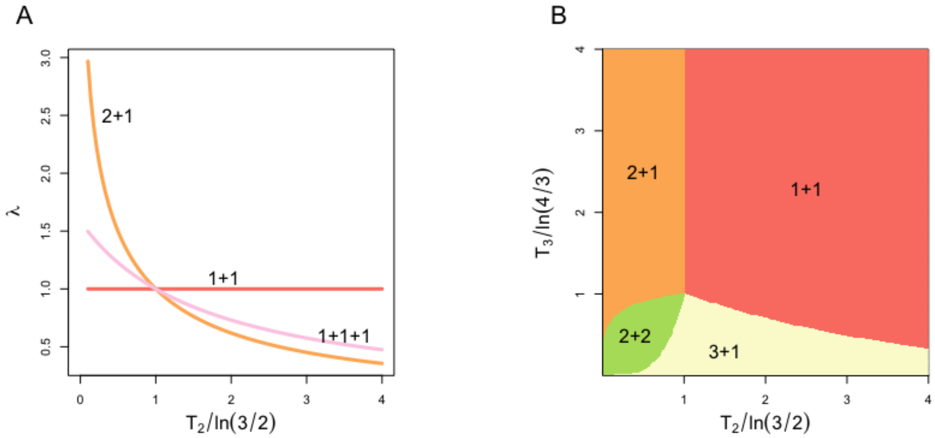

Fig 2. Growth rates and optimal life cycles in homogeneous groups on the condition of $n \leq 3$ and $n \leq 4$, respectively. A) describes the growth rates of life cycles when $n \leq 3$ i.e. 1+1, 1+1+1 and 2+1. **B**) shows the optimal life cycle when $n \leq 4$ with respect to T_2 and T_3. In both situations, T_i is the size increment time and we set $T_1 = \ln(2)$ for convenience.

Q corresponding to these life cycles are

$$\begin{aligned} Q_{1+1} &= \left(2e^{-\lambda T_1}\right), \\ Q_{2+1} &= \begin{pmatrix} e^{-\lambda(T_1+T_2)} & e^{-\lambda(T_1+T_2)} \\ e^{-\lambda T_2} & e^{-\lambda T_2} \end{pmatrix}, \\ Q_{1+1+1} &= \left(3e^{-\lambda(T_1+T_2)}\right). \end{aligned} \tag{29}$$

According to Eq 4, the growth rate of each life cycle are given by the solutions of

$$2e^{-\lambda_{1+1}T_1} - 1 = 0, \tag{30}$$

$$e^{-\lambda_{2+1}(T_1+T_2)} + e^{-\lambda_{2+1}T_2} - 1 = 0, \tag{31}$$

$$3e^{-\lambda_{1+1+1}(T_1+T_2)} - 1 = 0, \tag{32}$$

where λ_{1+1}, λ_{2+1} and λ_{1+1+1} are the growth rate of 1+1, 2+1 and 1+1+1, respectively, see Fig 2A. For small T_2, the largest growth rate is achieved by 2+1 life cycle. In this case, bi-cellular groups produce offspring cells faster than independent cells. Consequently, the life cycle 2+1, which allows production of bi-cellular groups (unlike unicellular life cycle 1+1) and preserving one offspring group in the most productive bi-cellular state (unlike 1+1+1) is most successful in growth competition. In the opposite limit of large T_2, the life cycle 1+1 leads to the largest population growth rate. In this case, independent cells are better off than bi-cellular groups. Thus, the best reproductive strategy is to avoid the growth to bi-cellular state, which can only be achieved with a single life cycle 1+1. In both situations of T_2, the growth rate of 1+1+1 is always between that of 1+1 and 2+1.

For the next scenario, we increase the maximal size of the group to three cells. This allows four new life cycles: 3+1, 2+2, 2+1+1 and 1+1+1+1. Their growth rates are given by the solutions of

$$e^{-\lambda_{3+1}(T_1+T_2+T_3)} + e^{-\lambda_{3+1}T_3} - 1 = 0 \tag{33}$$

$$2e^{-\lambda_{2+2}(T_2+T_3)} - 1 = 0 \tag{34}$$

$$2e^{-\lambda_{2+1+1}(T_1+T_2+T_3)} + e^{-\lambda_{2+1+1}(T_2+T_3)} - 1 = 0 \tag{35}$$

$$4e^{-\lambda_{1+1+1+1}(T_1+T_2+T_3)} - 1 = 0, \tag{36}$$

For large T_3, the life cycles which do not produce slow-growing three-cellular groups have the highest growth rates. Therefore, for large T_3, the optimal life cycles are the same as ones presented in the previous paragraph. For small T_3, the life cycles capable of producing three-cellular groups gain an evolutionary advantage. Specifically, 2+2 achieves the maximum growth rate when both T_2 and T_3 are comparatively small. In this case, an independent cell is the least productive state, whereas 2+2 is the only life cycle not producing independent cells. Life cycle 3+1 leads to the largest growth rate if T_3 is small but T_2 is large. There, the three-cellular group stands out as the most productive state, and 3+1 is the only life cycle keeping it as one of its offspring groups. Similarly to the previous scenario, life cycles with more than two offspring: 1+1+1, 2+1+1, 1+1+1+1, are never optimal. An important exception to this is the point $T_1 = \ln(2)$, $T_2 = \ln(\frac{3}{2})$ and $T_3 = \ln(\frac{4}{3})$, where all seven life cycles lead to the same growth rate ($\lambda = 1$).

Previously, we considered another model of life cycles evolution (1). There, the growth of groups from size i to size $i + 1$ occurs spontaneously with rate ib_i. Therefore, in that model, the time between cell divisions varies between groups of the same size, in contrast to the scenario considered here, where this time is always equal to T_i. Despite the differences between two models, they both share a number of findings: existence of the neutral point, only binary fragmentation is evolutionarily optimal, same optimal life cycles in the limit cases. Therefore, these features, are independent from the model design.

References

1. Pichugin Y, Peña J, Rainey P, Traulsen A. Fragmentation modes and the evolution of life cycles. PLoS Computational Biology. 2017;13(11):e1005860.
