# Supplementary material for: Interacting cells driving the evolution of multicellular life cycles
Source: PLoS Comput Biol. 2019 May 14;15(5):e1006987. doi: 10.1371/journal.pcbi.1006987 (PMC6534324; doi:10.1371/journal.pcbi.1006987)
Supplement: S4 Appendix — (PDF) [file pcbi.1006987.s004.pdf]

Interacting cells driving the evolution of multicellular life cycles

Yuanxiao Gao[1], Arne Traulsen[1], Yuriy Pichugin[1*]

1 Max Planck Institute for Evolutionary Biology, August-Thienemann-Str. 2, 24306 Plön, Germany

* pichugin@evolbio.mpg.de

Supporting information

S4 Appendix.

Calculation of growth rates λ for life cycles of heterogeneous groups. To show how our approach can can be used in the case of heterogeneous groups, consider the simplest unicellular life cycle 1+1. There are two types of offspring possible: independent A and B cells, so the matrix Q has dimensions 2 by 2. When a cell divides into two, three outcomes are possible: no cell, one cell, or both daughter cells change the phenotype. Since the developmental trajectory ends after the first division, there are only six developmental trajectories possible for this life cycle, see Fig 3.

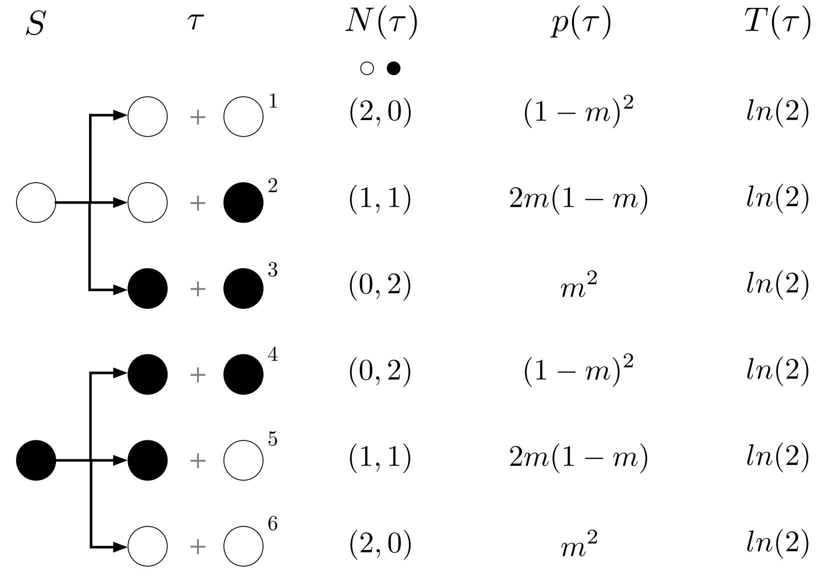

Fig 3. The full set of developmental trajectories in the life cycle 1+1. Here, the white and black circles denote A type cell and B type cell respectively.

To construct the matrix Q, we need to obtain the distribution of offspring (N_i), the probability of realization (p) and total developmental time (T) for each trajectory. Offspring distributions are apparent from Fig 3. The probability of each trajectory can be directly computed from the phenotype switch probability m. The developmental time is $T = T_1 = \ln(2)$ for each trajectory here. Therefore, the elements of Q are given by

$$Q_{11} = 2(1-m)^2 e^{-\lambda ln 2} \qquad \longleftarrow \tau_1$$

$$
\begin{aligned}
&+ 2m(1-m)e^{-\lambda ln2} &\longleftarrow \tau_2,\\
Q_{12} =&2m(1-m)^2e^{-\lambda ln2} &\longleftarrow \tau_2\\
&+ 2me^{-\lambda ln2} &\longleftarrow \tau_3,\\
Q_{21} =&2(1-m)e^{-\lambda ln2} &\longleftarrow \tau_5\\
&+ 2m^2e^{-\lambda ln2} &\longleftarrow \tau_6,\\
Q_{22} =&2(1-m)^2e^{-\lambda ln2} &\longleftarrow \tau_4\\
&+ 2m(1-m)e^{-\lambda ln2} &\longleftarrow \tau_5,
\end{aligned}
$$

where arrows indicate the index of the developmental trajectory contributing a given term. Solution of the Eq (18) leads to $\lambda_{1+1} = 1$ in the life cycle 1+1.

Next, consider the life cycle 1+1+1. There are still two types of offspring possible: independent A and B cells, such that the matrix Q has dimensions 2 by 2. However, the life cycle requires two divisions to complete, so the number of possible developmental trajectories is increased to 20. Also, interactions play a role during the second division, so the probabilities p and developmental times T are more complicated, see Fig 4.

The elements of matrix Q are given by

$$
\begin{aligned}
Q_{11} =&3n^4e^{-\lambda(t_{[1,0]}+t_{[2,0]})} &\longleftarrow \tau_1\\
&+ 4mn^3e^{-\lambda(t_{[1,0]}+t_{[2,0]})} &\longleftarrow \tau_2\\
&+ m^2n^2e^{-\lambda(t_{[1,0]}+t_{[2,0]})} &\longleftarrow \tau_3\\
&+ 6m^3nP^B_{1+1}e^{-\lambda(t_{[1,0]}+t_{[1,1]})} &\longleftarrow \tau_4\\
&+ 4mn^2(nP^A_{1+1} + 2mP^B_{1+1})e^{-\lambda(t_{[1,0]}+t_{[1,1]})} &\longleftarrow \tau_5\\
&+ 2mn^2(2mP^A_{1+1} + nP^B_{1+1})e^{-\lambda(t_{[1,0]}+t_{[1,1]})} &\longleftarrow \tau_6\\
&+ 2m^4e^{-\lambda(t_{[1,0]}+t_{[0,2]})} &\longleftarrow \tau_8\\
&+ 2m^3ne^{-\lambda(t_{[1,0]}+t_{[0,2]})} &\longleftarrow \tau_9\\
Q_{12} =&2mn^3e^{-\lambda(t_{[1,0]}+t_{[2,0]})} &\longleftarrow \tau_2\\
&+ 2m^2n^2e^{-\lambda(t_{[1,0]}+t_{[2,0]})} &\longleftarrow \tau_3\\
&+ 2mn^2(nP^A_{1+1} + 2mP^B_{1+1})e^{-\lambda(t_{[1,0]}+t_{[1,1]})} &\longleftarrow \tau_5\\
&+ 4mn^2(2mP^A_{1+1} + nP^B_{1+1})e^{-\lambda(t_{[1,0]}+t_{[1,1]})} &\longleftarrow \tau_6\\
&+ 6m^3nP^A_{1+1}e^{-\lambda(t_{[1,0]}+t_{[1,1]})} &\longleftarrow \tau_7\\
&+ m^4e^{-\lambda(t_{[1,0]}+t_{[0,2]})} &\longleftarrow \tau_8\\
&+ 4m^3ne^{-\lambda(t_{[1,0]}+t_{[0,2]})} &\longleftarrow \tau_9\\
&+ 3m^2n^2e^{-\lambda(t_{[1,0]}+t_{[0,2]})} &\longleftarrow \tau_{10}\\
Q_{21} =&2mn^3e^{-\lambda(t_{[0,1]}+t_{[0,2]})} &\longleftarrow \tau_{12}\\
&+ 2m^2n^2e^{-\lambda(t_{[0,1]}+t_{[0,2]})} &\longleftarrow \tau_{13}\\
&+ 2mn^2(2mP^A_{1+1} + nP^B_{1+1})e^{-\lambda(t_{[0,1]}+t_{[1,1]})} &\longleftarrow \tau_{15}\\
&+ 4mn^2(nP^A_{1+1} + 2mP^B_{1+1})e^{-\lambda(t_{[0,1]}+t_{[1,1]})} &\longleftarrow \tau_{16}\\
&+ 6m^3nP^B_{1+1}e^{-\lambda(t_{[0,1]}+t_{[1,1]})} &\longleftarrow \tau_{17}\\
&+ m^4e^{-\lambda(t_{[0,1]}+t_{[2,0]})} &\longleftarrow \tau_{18}\\
&+ 4m^3ne^{-\lambda(t_{[0,1]}+t_{[2,0]})} &\longleftarrow \tau_{19}
\end{aligned}
$$

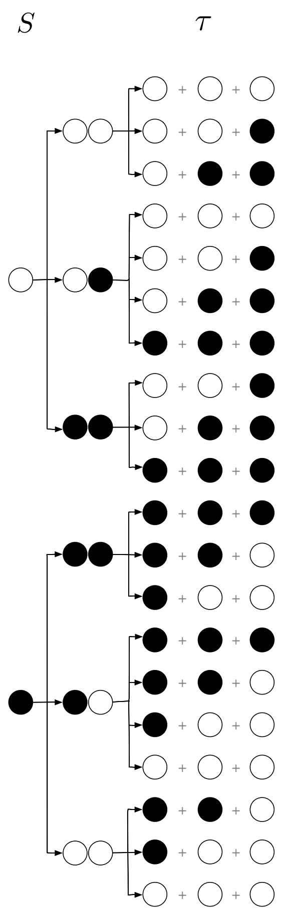

$N(\tau)$ ○ ●	$p(\tau)$	$T(\tau)$
$(3,0)$	n^4	$t_{[1,0]}+t_{[2,0]}$
$(2,1)$	$2mn^3$	$t_{[1,0]}+t_{[2,0]}$
$(1,2)$	m^2n^2	$t_{[1,0]}+t_{[2,0]}$
$(3,0)$	$2m^3nP^B_{[1,1]}$	$t_{[1,0]}+t_{[1,1]}$
$(2,1)$	$2mn(n^2P^A_{[1,1]}+2mnP^B_{[1,1]})$	$t_{[1,0]}+t_{[1,1]}$
$(1,2)$	$2mn(2mnP^A_{[1,1]}+n^2P^B_{[1,1]})$	$t_{[1,0]}+t_{[1,1]}$
$(0,3)$	$2m^3nP^A_{[1,1]}$	$t_{[1,0]}+t_{[1,1]}$
$(2,1)$	m^4	$t_{[1,0]}+t_{[0,2]}$
$(1,2)$	$2m^3n$	$t_{[1,0]}+t_{[0,2]}$
$(0,3)$	m^2n^2	$t_{[1,0]}+t_{[0,2]}$
$(0,3)$	n^4	$t_{[0,1]}+t_{[0,2]}$
$(1,2)$	$2mn^3$	$t_{[0,1]}+t_{[0,2]}$
$(2,1)$	m^2n^2	$t_{[0,1]}+t_{[0,2]}$
$(0,3)$	$2m^3nP^A_{[1,1]}$	$t_{[0,1]}+t_{[1,1]}$
$(1,2)$	$2mn(2mnP^A_{[1,1]}+n^2P^B_{[1,1]})$	$t_{[0,1]}+t_{[1,1]}$
$(2,1)$	$2mn(n^2P^A_{[1,1]}+2mnP^B_{[1,1]})$	$t_{[0,1]}+t_{[1,1]}$
$(3,0)$	$2m^3nP^B_{[1,1]}$	$t_{[0,1]}+t_{[1,1]}$
$(1,2)$	m^4	$t_{[0,1]}+t_{[2,0]}$
$(2,1)$	$2m^3n$	$t_{[0,1]}+t_{[2,0]}$
$(3,0)$	m^2n^2	$t_{[0,1]}+t_{[2,0]}$

Fig 4. The full set of developmental trajectories in the life cycle 1+1+1. White circles represent A type cells and black circles represent B type cells. For simplicity of notation, we use $n = 1 - m$, $t_{[i,j]}$ is the time before the next cell division for a complex with i A type cells and j B type cells to divide; and $P^A_{[1,1]} = \frac{1}{2} + w\frac{b-c}{4}$ and $P^B_{[1,1]} = \frac{1}{2} - w\frac{b-c}{4}$, see details in the model section of the main text.

$$
\begin{aligned}
&+ 3m^2n^2e^{-\lambda(t_{[0,1]}+t_{[2,0]})} && \longleftarrow \tau_{20}\\
Q_{22} =& 3n^4e^{-\lambda(t_{[0,1]}+t_{[0,2]})} && \longleftarrow \tau_{11}\\
&+ 4mn^3e^{-\lambda(t_{[0,1]}+t_{[0,2]})} && \longleftarrow \tau_{12}\\
&+ m^2n^2e^{-\lambda(t_{[0,1]}+t_{[0,2]})} && \longleftarrow \tau_{13}\\
&+ 6m^3nP^A_{1+1}e^{-\lambda(t_{[0,1]}+t_{[1,1]})} && \longleftarrow \tau_{14}\\
&+ 4mn^2(2mP^A_{1+1}+nP^B_{1+1})e^{-\lambda(t_{[0,1]}+t_{[1,1]})} && \longleftarrow \tau_{15}\\
&+ 2mn^2(nP^A_{1+1}+2mP^B_{1+1})e^{-\lambda(t_{[0,1]}+t_{[1,1]})} && \longleftarrow \tau_{16}\\
&+ 2m^4e^{-\lambda(t_{[0,1]}+t_{[2,0]})} && \longleftarrow \tau_{18}\\
&+ 2m^3ne^{-\lambda(t_{[0,1]}+t_{[2,0]})} && \longleftarrow \tau_{19}
\end{aligned}
$$

Here, $P^A_{1+1} = \frac{1}{2} + w\frac{b-c}{4}$, $P^B_{1+1} = \frac{1}{2} - w\frac{b-c}{4}$, $t_{[1,0]} = t_{[1,0]} = ln2$, $t_{[2,0]} = ln\frac{3}{2}(1 - wa)$, $t_{[1,1]} = ln2 + ln\frac{3}{2}(1 - w\frac{b+c}{2})$ and $t_{[2,0]} = ln\frac{3}{2}(1 - wd)$. Arrows indicate the contributions of

each developmental trajectory to Q_{ij}.

The solution of Eq (18) for life cycle 1+1+1 yields

$$\lambda_{1+1+1} = 1 + w\frac{\ln(3/2)}{\ln(9)}(a+d)\left((1-2m+2m^2) + 2m(1-m)\frac{b+c}{a+d}\right). \tag{37}$$

Our final example is the life cycle 2+1, where groups grow to three cells and fragment into a bi-cellular group and an independent cell. Here, five offspring types are possible: independent cells could be either A or B type and the bi-cellular group could have composition AA, AB, or BB, see Figs. 5, 6. Therefore, Q is 5×5 matrix. There are 48 developmental programs possible and we refrain from showing here how elements of matrix Q are constructed in this case. The solution of the Eq (18) for life cycle 2+1 yields

$$\lambda_{2+1} = 1 + w\frac{3\ln(3/2)}{2(5+8m)\ln(27/4)}(a+d)\left((5-6m+10m^2) + 2m(7-5m)\frac{b+c}{a+d}\right). \tag{38}$$

For the life cycles 1+1+1+1 and 2+2, we just list the growth rate λ

$$\begin{aligned}\lambda_{1+1+1+1} =& 1 + w\frac{1}{12\ln(2)}(a+d)\left(3\ln(2) + (1-\frac{b+c}{a+d})\right.\\ &\left.\left(-2m(5\ln(2)-\ln(3)) + 2m^2(11\ln(2)-4\ln(3)) - 8m^3(2\ln(2)-\ln(3))\right)\right),\end{aligned} \tag{39}$$

$$\begin{aligned}\lambda_{2+2} =& 1 + w\frac{1}{4(8m^2-9m-2)\ln(2)}(a+d)\left(-4\ln(2) + m(\ln(3)-\ln(2)) - 4m^2\ln(3)\right.\\ &\left.+4m^3\ln(3) + \frac{b+c}{a+d}(-m(17\ln(2)+\ln(3)) + 4m^2(4\ln(2)-\ln(3)) - 4m^3\ln(3))\right).\end{aligned} \tag{40}$$

For more complex life cycles, the analytical expressions are too large to be meaningful by naked eye analysis. Therefore, we used a combination of analytical and numerical approaches. After the linearisation with respect to w, the growth rate for any life cycle has the form

$$\lambda_{x_1+\ldots+x_n} = 1 + w\frac{1}{P_1(m)}(a+d)\left(P_2(m) + \frac{b+c}{a+d}P_3(m)\right), \tag{41}$$

where $P_1(m), P_2(m), P_3(m)$ are some polynomials of m of the finite power. We obtained exact expressions for these polynomials using the symbolic algebra software. However, the tracking of all developmental programs means that the computation load grows exponentially with the maximal group size M. For life cycles, such as 3+2+1+1, computation of the polynomials required an extraordinary amount of RAM ($>$ 70 Gb) and the outcome is neither human-tractable nor even printable. This memory constraints is the factor, limiting the maximal group size considered to $M = 7$. Therefore, in our study, we only stored the numerical values of the coefficients of m in $P_1(m), P_2(m), P_3(m)$ and used them to compute λ. With this approach, we are able to compute numerical values of λ with very high accuracy, even if traditional closed form solutions are unavailable.

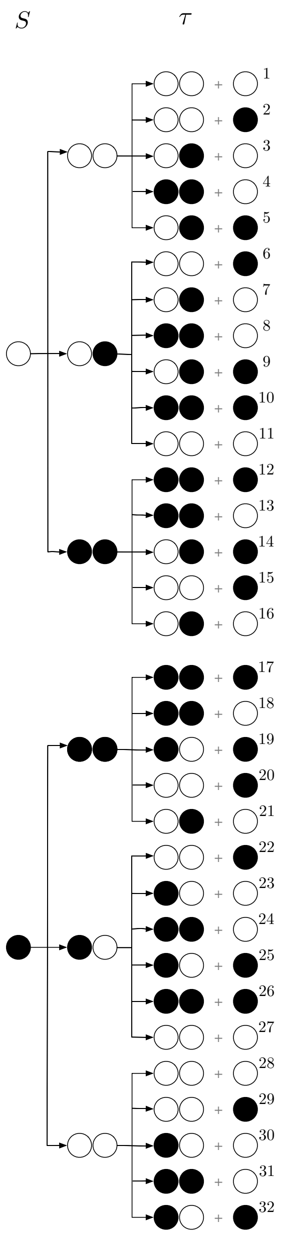

τ	$N(\tau)$ ○ ● ○○ ○● ●●	$p(\tau)$	$T(\tau)$
1	$(1,0,1,0,0)$	n^4	$t_{[1,0]}+t_{[2,0]}$
2	$(0,1,1,0,0)$	$2/3\ mn^3$	$t_{[1,0]}+t_{[2,0]}$
3	$(1,0,0,1,0)$	$4/3\ mn^3$	$t_{[1,0]}+t_{[2,0]}$
4	$(1,0,0,0,1)$	$1/3\ m^2n^2$	$t_{[1,0]}+t_{[2,0]}$
5	$(0,1,0,1,0)$	$2/3\ m^2n^2$	$t_{[1,0]}+t_{[2,0]}$
6	$(0,1,1,0,0)$	$2mn(1/3\ n^2P^A_{[1,1]}+2/3\ mnP^B_{[1,1]})$	$t_{[1,0]}+t_{[1,1]}$
7	$(1,0,0,1,0)$	$2mn(2/3\ n^2P^A_{[1,1]}+4/3\ mnP^B_{[1,1]})$	$t_{[1,0]}+t_{[1,1]}$
8	$(1,0,0,0,1)$	$2mn(2/3\ mnP^A_{[1,1]}+1/3\ n^2P^B_{[1,1]})$	$t_{[1,0]}+t_{[1,1]}$
9	$(0,1,0,1,0)$	$2mn(4/3\ mnP^A_{[1,1]}+2/3\ n^2P^B_{[1,1]})$	$t_{[1,0]}+t_{[1,1]}$
10	$(0,1,0,0,1)$	$2m^3nP^A_{[1,1]}$	$t_{[1,0]}+t_{[1,1]}$
11	$(1,0,1,0,0)$	$2m^3nP^B_{[1,1]}$	$t_{[1,0]}+t_{[1,1]}$
12	$(0,1,0,0,1)$	m^2n^2	$t_{[1,0]}+t_{[0,2]}$
13	$(1,0,0,0,1)$	$2/3\ m^3n$	$t_{[1,0]}+t_{[0,2]}$
14	$(0,1,0,1,0)$	$4/3\ m^3n$	$t_{[1,0]}+t_{[0,2]}$
15	$(0,1,1,0,0)$	$1/3\ m^4$	$t_{[1,0]}+t_{[0,2]}$
16	$(1,0,0,1,0)$	$2/3\ m^4$	$t_{[1,0]}+t_{[0,2]}$
17	$(0,1,0,0,1)$	n^4	$t_{[0,1]}+t_{[0,2]}$
18	$(1,0,0,0,1)$	$2/3\ mn^3$	$t_{[0,1]}+t_{[0,2]}$
19	$(0,1,0,1,0)$	$4/3\ mn^3$	$t_{[0,1]}+t_{[0,2]}$
20	$(0,1,1,0,0)$	$1/3\ m^2n^2$	$t_{[0,1]}+t_{[0,2]}$
21	$(1,0,0,1,0)$	$2/3\ m^2n^2$	$t_{[0,1]}+t_{[0,2]}$
22	$(0,1,1,0,0)$	$2mn(1/3\ n^2P^A_{[1,1]}+2/3\ mnP^B_{[1,1]})$	$t_{[0,1]}+t_{[1,1]}$
23	$(1,0,0,1,0)$	$2mn(2/3\ n^2P^A_{[1,1]}+4/3\ mnP^B_{[1,1]})$	$t_{[0,1]}+t_{[1,1]}$
24	$(1,0,0,0,1)$	$2mn(2/3\ mnP^A_{[1,1]}+1/3\ n^2P^B_{[1,1]})$	$t_{[0,1]}+t_{[1,1]}$
25	$(0,1,0,1,0)$	$2mn(4/3\ mnP^A_{[1,1]}+2/3\ n^2P^B_{[1,1]})$	$t_{[0,1]}+t_{[1,1]}$
26	$(0,1,0,0,1)$	$2m^3nP^A_{[1,1]}$	$t_{[0,1]}+t_{[1,1]}$
27	$(1,0,1,0,0)$	$2m^3nP^B_{[1,1]}$	$t_{[0,1]}+t_{[1,1]}$
28	$(1,0,1,0,0)$	m^2n^2	$t_{[0,1]}+t_{[2,0]}$
29	$(0,1,1,0,0)$	$2/3\ m^3n$	$t_{[0,1]}+t_{[2,0]}$
30	$(1,0,0,1,0)$	$4/3\ m^3n$	$t_{[0,1]}+t_{[2,0]}$
31	$(1,0,0,0,1)$	$1/3\ m^4$	$t_{[0,1]}+t_{[2,0]}$
32	$(0,1,0,1,0)$	$2/3\ m^4$	$t_{[0,1]}+t_{[2,0]}$

Fig 5. The developmental trajectories of the single A-cell and B-cell in the life cycle 2+1. White circles represent A type cells and black circles represent B type cells. For simplicity of notation, we use: $n = 1 - m$; $t_{[i,j]}$ is the time before the next cell division for a complex with i A type cells and j B type cells to divide; and $P^A_{[1,1]} = \frac{1}{2} + w\frac{b-c}{4}$ and $P^B_{[1,1]} = \frac{1}{2} - w\frac{b-c}{4}$, see details in the model section of the main text.

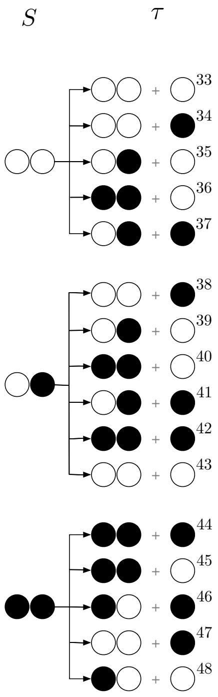

S / τ	$N(\tau)$ (○ ● ○○ ○● ●●)	$p(\tau)$	$T(\tau)$
33	$(1,0,1,0,0)$	n^2	$t_{[2,0]}$
34	$(0,1,1,0,0)$	$2/3\ mn$	$t_{[2,0]}$
35	$(1,0,0,1,0)$	$4/3\ mn$	$t_{[2,0]}$
36	$(1,0,0,0,1)$	$1/3\ m^2$	$t_{[2,0]}$
37	$(0,1,0,1,0)$	$2/3\ m^2$	$t_{[2,0]}$
38	$(0,1,1,0,0)$	$1/3\ n^2P^A_{[1,1]} + 2/3\ mnP^B_{[1,1]}$	$t_{[1,1]}$
39	$(1,0,0,1,0)$	$2/3\ n^2P^A_{[1,1]} + 4/3\ mnP^B_{[1,1]}$	$t_{[1,1]}$
40	$(1,0,0,0,1)$	$2/3\ mnP^A_{[1,1]} + 1/3\ n^2P^B_{[1,1]}$	$t_{[1,1]}$
41	$(0,1,0,1,0)$	$4/3\ mnP^A_{[1,1]} + 2/3\ n^2P^B_{[1,1]}$	$t_{[1,1]}$
42	$(0,1,0,0,1)$	$m^2P^A_{[1,1]}$	$t_{[1,1]}$
43	$(1,0,1,0,0)$	$m^2P^B_{[1,1]}$	$t_{[1,1]}$
44	$(0,1,0,0,1)$	n^2	$t_{[0,2]}$
45	$(1,0,0,0,1)$	$2/3\ mn$	$t_{[0,2]}$
46	$(0,1,0,1,0)$	$4/3\ mn$	$t_{[0,2]}$
47	$(0,1,1,0,0)$	$1/3\ m^2$	$t_{[0,2]}$
48	$(1,0,0,1,0)$	$2/3\ m^2$	$t_{[0,2]}$

Fig 6. The developmental programs of bicellular newborn groups cells in the life cycle 2+1. White circles represent A type cells and black circles represent B type cells. For simplicity of notation, we use: $n = 1 - m$; $t_{[i,j]}$ is the time before the next cell division for a complex with i A type cells and j B type cells to divide; and $P^A_{[1,1]} = \frac{1}{2} + w\frac{b-c}{4}$ and $P^B_{[1,1]} = \frac{1}{2} - w\frac{b-c}{4}$, see details in the model section of the main text.
