# Supplementary material for: Interacting cells driving the evolution of multicellular life cycles
Source: PLoS Comput Biol. 2019 May 14;15(5):e1006987. doi: 10.1371/journal.pcbi.1006987 (PMC6534324; doi:10.1371/journal.pcbi.1006987)
Supplement: S5 Appendix — (PDF) [file pcbi.1006987.s005.pdf]

Interacting cells driving the evolution of multicellular life cycles

Yuanxiao Gao[1], Arne Traulsen[1], Yuriy Pichugin[1*]

1 Max Planck Institute for Evolutionary Biology, August-Thienemann-Str. 2, 24306 Plön, Germany

* pichugin@evolbio.mpg.de

Supporting information

S5 Appendix.

Profiles of growth rates of the life cycles. In this appendix, we present profiles of growth rates at different conditions. The growth rate is determined by three parameters: ψ, ϕ and m. The greatest diversity of evolutionarily optimal life cycles is observed at $\psi > 0$ and small m, see Fig 7. In this case, we observed two clusters of life cycles, where life cycles behave quite similar. One cluster contain the multiple fission life cycles such as 1+1, 1+1+1. The second cluster is the group propagules life cycles such as 3+2, 4+3. The slope of multiple fission life cycles are increasing with colony size, see Fig 7C. More similar growth rate patterns are observed for the group propagules life cycles, which have identical growth rates at $\phi = 1$, see Fig 7B. Most other life cycles are between the area of multiple fission life cycles and group propagules life cycles, which can never be optimal. For $\psi > 0$ and large m, only one multiple fission life cycle, 1+1+1, is evolutionary optimal, see Fig 8A. Its area of optimality is located between unicellularity (1+1) at large negative ϕ and binary fragmentation with multicellular propagules (2+2 and 4+3) at large positive ϕ. Considering the dependence of growth rate from the phenotype switching probability m, we found that at $\phi \gg 1$, growth rate profiles are concave functions of m, see Fig 8B. Growth rates of most life cycles are generally bound between binary fragmentation with multicellular propagules (such as 2+2 and 4+3) and multiple fragmentation with unicellular propagules (such as 1+1+1 and 1+1+1+1). For $\phi \ll -1$, the pattern is very similar, with an exception, that growth rate profiles are convex, instead of concave, and the hierarchy of life cycles is reversed, see Fig 8C. This leads to the great diversity of evolutionary optimal life cycles at $\phi < 0$ and small m (including also transitional life cycles 2+1 and 2+1+1, as well as binary fragmentation 2+2), see Fig 8D.

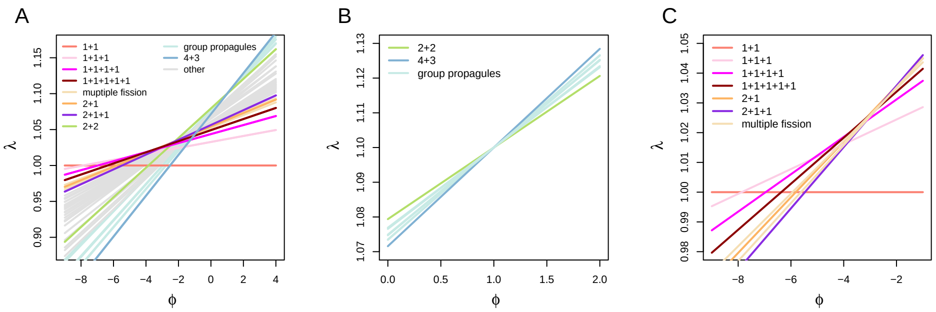

Fig 7. The growth rates of the considered life cycles as a function of ϕ for $\psi > 0$. Panel A: according to the weak selection approximation, growth rates λ are linear functions of ϕ. For all life cycles, the slope of the line is non-negative, thus, life cycles with smaller slope dominate at $\phi \ll -1$ (1+1 has slope zero) and life cycles with larger slope dominate at $\phi \gg 1$ (4+3 has the largest slope for $M \leq 7$). Panel B: all life cycles with multicellular offspring share the same growth rate at $\phi = 1$ ($\phi = -1$ under $\psi < 0$). Panel C: a sequence of multiple fission life cycles is optimal at the negative ϕ. At all panels $m = 0.06$. In all panels, multiple fission includes 1+1+1+1+1+1, 1+1+1+1+1+1+1; group fission includes 3+2, 3+3, 2+2+2, 4+2, 5+2.

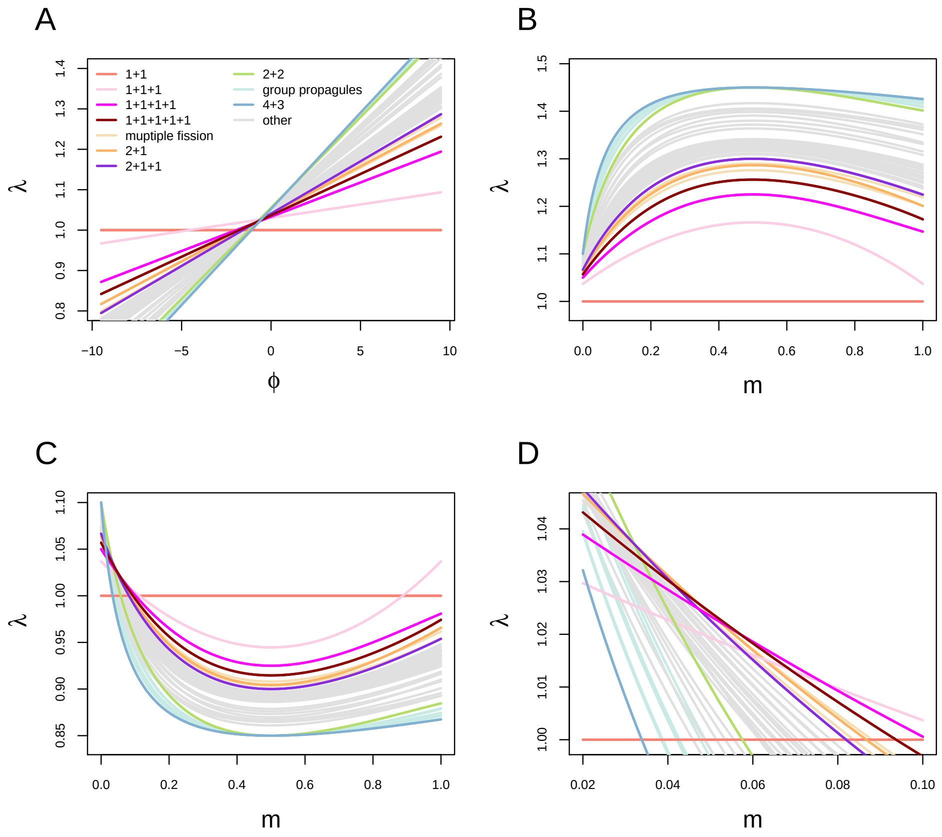

Fig 8. Multiple life cycles are optimal for $\psi > 0$. A Growth rates of all considered life cycles as function of ϕ at $m = 0.9$ (cf. Fig 4A for $m = 0.06$). **B** Growth rates of all considered life cycles as function of m at $\phi = 8$. **C** Growth rates of all considered life cycles as function of m at $\phi = -4$. **D** Detailed view of the panel C in the range of small m showing that large number of evolutionary optimal life cycles at different m.

At the negative ψ, only two life cycles were found to be optimal, see Fig 9. The shape of individual growth rate profiles remain similar to the case of positive ϕ but the relative position changes significantly. Thus, the spectrum of observed life cycles is much less diverse.

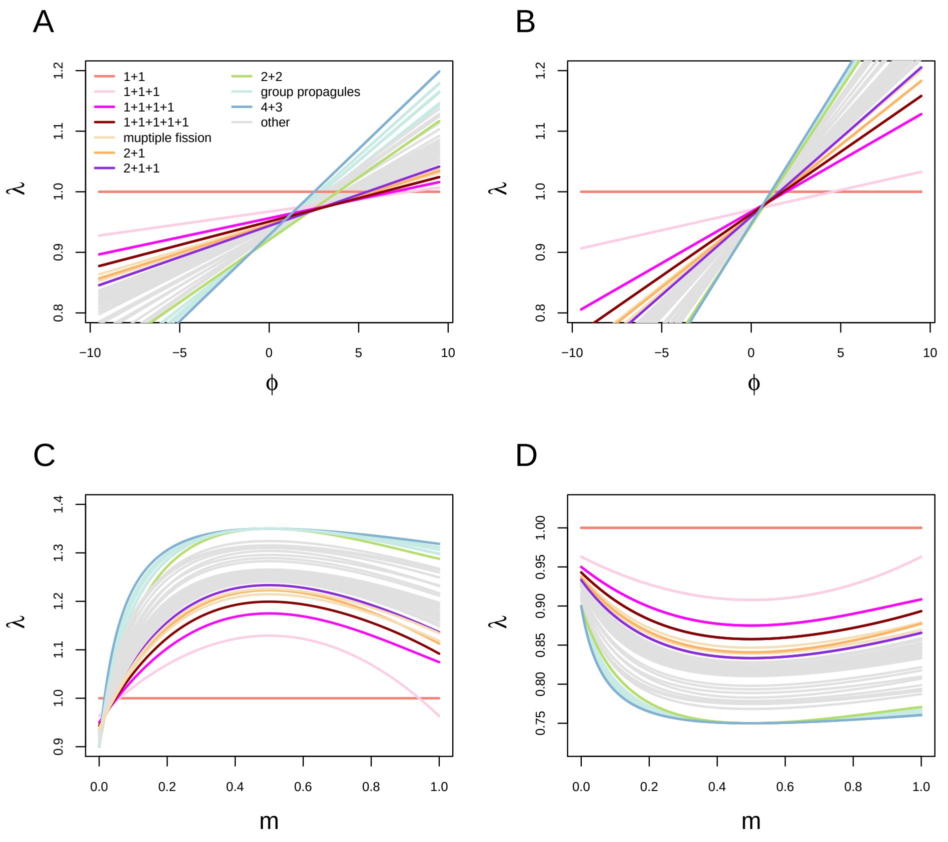

Fig 9. Only two life cycles are optimal for $\psi < 0$. A Growth rates of all considered life cycles as function of ϕ at $m = 0.06$. **B** Growth rates of all considered life cycles as function of ϕ at $m = 0.9$. **C** Growth rates of all considered life cycles as function of m at $\phi = 8$. **D** Growth rates of all considered life cycles as function of m at $\phi = -4$.
