# Supplementary material for: Interacting cells driving the evolution of multicellular life cycles
Source: PLoS Comput Biol. 2019 May 14;15(5):e1006987. doi: 10.1371/journal.pcbi.1006987 (PMC6534324; doi:10.1371/journal.pcbi.1006987)
Supplement: S6 Appendix — (PDF) [file pcbi.1006987.s006.pdf]

Interacting cells driving the evolution of multicellular life cycles

Yuanxiao Gao[1], Arne Traulsen[1], Yuriy Pichugin[1*]

1 Max Planck Institute for Evolutionary Biology, August-Thienemann-Str. 2, 24306 Plön, Germany

* pichugin@evolbio.mpg.de

Supporting information

S6 Appendix.

Optimal life cycles landscape under the self-interaction game. In our model setting, we set the payoff of single cells to zero based on the assumption that no other cells can impact their strategies. While, theoretically single cells can also play self-interaction games to get payoff based on their cell types. Intuitively, the self-interaction game would produce the same results as the non self-interaction game, as in which only the final synergistic or antagonistic effects can really impact the outcome. To check this idea, we set the payoff of cells in a cluster to

$$\alpha_{[i,j]} = \frac{ia + jb}{i + j},$$
$$\beta_{[i,j]} = \frac{ic + jd}{i + j}, \qquad (8)$$

where $\alpha_{[i,j]}$ and $\beta_{[i,j]}$ are the average payoff of A type cells and B type cells in a group of i A-cells and j B-cells, respectively. This payoff definition allows the single cells also have non zero payoff values i.e. payoff a for A cell type and d for B cell type. Meanwhile, all other settings in the model are unchanged. Then, we investigate the optimal life cycles for population with colony size M less than seven. The results are pretty similar between the non self-interaction game (see Fig 4) and the self-interaction game (see Fig 10).

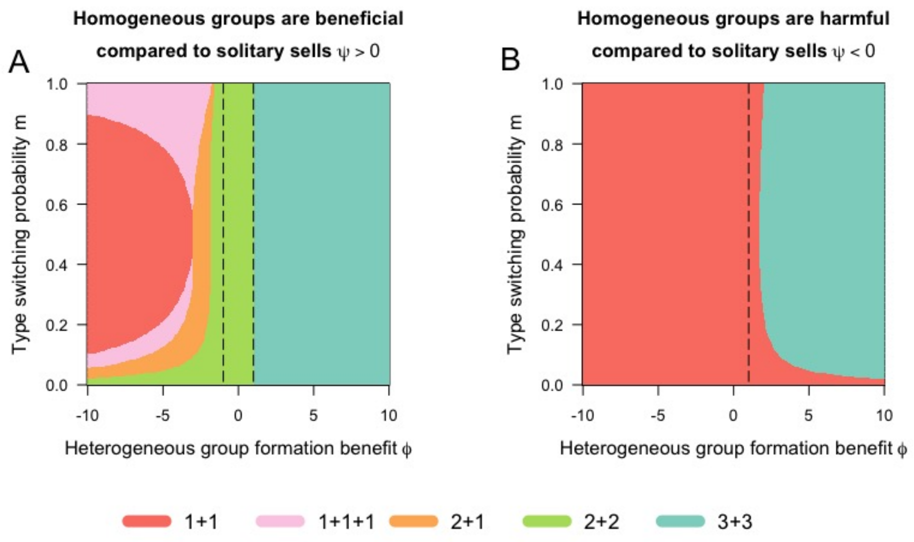

Fig 10. Similar optimal life cycles under the self-interaction game compared with the no self-interaction game. While we have only 5 optimal life cycles in this case, in general the results are very similar, with a large number of life cycles emerging only for $\psi > 0$ and $\phi < -1$. **A** Optimal life cycles for $\psi > 0$ under the self-interaction game. Dashed lines are $\phi = -1$ and $\phi = 1$, respectively. **B** Optimal life cycles for $\psi < 0$ under the self-interaction game. Dashed line is $\phi = 1$.
